# Supplementary material for: Conscious Sedation for Dental Treatments in Subjects with Intellectual Disability: A Systematic Review and Meta-Analysis
Source: Int J Environ Res Public Health. 2023 Jan 18;20(3):1779. doi: 10.3390/ijerph20031779 (PMC9914902; doi:10.3390/ijerph20031779)
Supplement: Supplementary file 1 [file ijerph-20-01779-s001.zip › ijerph-2148944-supplementary.docx]

**Supplementary files for “Efficacy of conscious sedation for performing dental treatments in subjects with intellectual disability- a systematic review and meta-analysis”**

Table S1. Preferred Reporting Items for Systematic reviews and Meta-Analyses extension (PRISMA) Checklist

| **Section/topic** | **#** | **Checklist item** | **Reported on page #** |
| --- | --- | --- | --- |
| **TITLE** | | |  |
| Title | 1 | Identify the report as a systematic review, meta-analysis, or both. | 1 |
| **ABSTRACT** | | |  |
| Structured summary | 2 | Provide a structured summary including, as applicable: background; objectives; data sources; study eligibility criteria, participants, and interventions; study appraisal and synthesis methods; results; limitations; conclusions and implications of key findings; systematic review registration number. | 1 |
| **INTRODUCTION** | | |  |
| Rationale | 3 | Describe the rationale for the review in the context of what is already known. | 1,2 |
| Objectives | 4 | Provide an explicit statement of questions being addressed with reference to participants, interventions, comparisons, outcomes, and study design (PICOS). | 2 |
| **METHODS** | | |  |
| Protocol and registration | 5 | Indicate if a review protocol exists, if and where it can be accessed (e.g., Web address), and, if available, provide registration information including registration number. | 2 |
| Eligibility criteria | 6 | Specify study characteristics (e.g., PICOS, length of follow-up) and report characteristics (e.g., years considered, language, publication status) used as criteria for eligibility, giving rationale. | 2,3 |
| Information sources | 7 | Describe all information sources (e.g., databases with dates of coverage, contact with study authors to identify additional studies) in the search and date last searched. | 3 |
| Search | 8 | Present full electronic search strategy for at least one database, including any limits used, such that it could be repeated. | 3,4 |
| Study selection | 9 | State the process for selecting studies (i.e., screening, eligibility, included in systematic review, and, if applicable, included in the meta-analysis). | 4 |
| Data collection process | 10 | Describe method of data extraction from reports (e.g., piloted forms, independently, in duplicate) and any processes for obtaining and confirming data from investigators. | 4,5 |
| Data items | 11 | List and define all variables for which data were sought (e.g., PICOS, funding sources) and any assumptions and simplifications made. | 2,3 |
| Risk of bias in individual studies | 12 | Describe methods used for assessing risk of bias of individual studies (including specification of whether this was done at the study or outcome level), and how this information is to be used in any data synthesis. | 5 |
| Summary measures | 13 | State the principal summary measures (e.g., risk ratio, difference in means). | 5 |
| Synthesis of results | 14 | Describe the methods of handling data and combining results of studies, if done, including measures of consistency (e.g., I^2^) for each meta-analysis. | 5 |
| Risk of bias across studies | 15 | Specify any assessment of risk of bias that may affect the cumulative evidence (e.g., publication bias, selective reporting within studies). | 5 |
| Additional analyses | 16 | Describe methods of additional analyses (e.g., sensitivity or subgroup analyses, meta-regression), if done, indicating which were pre-specified. | 5 |
| **RESULTS** | | |  |
| Study selection | 17 | Give numbers of studies screened, assessed for eligibility, and included in the review, with reasons for exclusions at each stage, ideally with a flow diagram. | 6 |
| Study characteristics | 18 | For each study, present characteristics for which data were extracted (e.g., study size, PICOS, follow-up period) and provide the citations. | 6,7 |
| Risk of bias within studies | 19 | Present data on risk of bias of each study and, if available, any outcome level assessment (see item 12). |  |
| Results of individual studies | 20 | For all outcomes considered (benefits or harms), present, for each study: (a) simple summary data for each intervention group (b) effect estimates and confidence intervals, ideally with a forest plot. |  |
| Synthesis of results | 21 | Present results of each meta-analysis done, including confidence intervals and measures of consistency. |  |
| Risk of bias across studies | 22 | Present results of any assessment of risk of bias across studies (see Item 15). |  |
| Additional analysis | 23 | Give results of additional analyses, if done (e.g., sensitivity or subgroup analyses, meta-regression [see Item 16]). |  |
| **DISCUSSION** | | |  |
| Summary of evidence | 24 | Summarize the main findings including the strength of evidence for each main outcome; consider their relevance to key groups (e.g., healthcare providers, users, and policy makers). |  |
| Limitations | 25 | Discuss limitations at study and outcome level (e.g., risk of bias), and at review-level (e.g., incomplete retrieval of identified research, reporting bias). |  |
| Conclusions | 26 | Provide a general interpretation of the results in the context of other evidence, and implications for future research. |  |
| **FUNDING** | | |  |
| Funding | 27 | Describe sources of funding for the systematic review and other support (e.g., supply of data); role of funders for the systematic review. |  |

Table S2. Data extraction form

| **Author** |  |  |  |
| --- | --- | --- | --- |
| **Title** |  |  |  |
| **Item Type** |  |  |  |
| **Journal** |  |  |  |
| **Country** |  |  |  |
| **Type of study** |  |  |  |
| **Number of patient-TOT.** |  |  |  |
| **Number of patient-TEST** |  |  |  |
| **Number of patient-CONTROL** |  |  |  |
| **Age rage (mean)** |  |  |  |
| **M/F** |  |  |  |
| **Type of disability** |  |  |  |
| **Evaluation scale adopted** |  |  |  |
| **ICDAS/dmft** |  |  |  |
| **Drugs administered-TEST** |  |  |  |
| **Drugs administered-CONTROL** |  |  |  |
| **Setting** |  |  |  |
| **Operator** |  |  |  |
| **Blinding** |  |  |  |
| **Follow up** |  |  |  |
| **Primary outcomes** |  |  |  |
| **Secondary outcomes** |  |  |  |
| **Sponsor** |  |  |  |
| **Results-TEST** |  |  |  |
| **TEST p-value** |  |  |  |
| **Results-CONTROL** |  |  |  |
| **CONTROL p-value** |  |  |  |
| **Database** |  |  |  |

Table S3. Excluded articles and reason for exclusion after title and abstract evaluation.

| **Title** | **Authors** | **Published Year** | **Journal** | **Reason of exclusion** |
| --- | --- | --- | --- | --- |
| **Dexmedetomidine Adjuvant Treatment for Depressed Patients Undergoing ECT** | A Alawi, M.S. | 2020 | ClinicalTrials.gov | Registration protocol |
| **Treatment modalities in a dental fear clinic and the relation with general psychopathology and oral health variables** | Aartman, I H A; De Jongh, A; Makkes, P C; Hoogstraten, J | 1999 | British Dental Journal | Different outcome |
| **Recovery following treatment of descending thoracic aortic disease. A comparison between endovascular repair and open surgery** | Aasland, J; Lundbom, J; Eide, T O; Ødegård, A; Aadahl, P; Romundstad, P R; Myhre, H O | 2005 | International Angiology | Search noise |
| **Caries experience, periodontal status, and metabolic morbidity in patients with psychiatric disorders** | Abdalla-Aslan, R; Findler, M; Zini, A; Almoznino, G | 2021 | Quintessence International | Search noise |
| **Ketamine Induces Lasting Antidepressant Effects by Modulating the NMDAR/CaMKII-Mediated Synaptic Plasticity of the Hippocampal Dentate Gyrus in Depressive Stroke Model** | Abdoulaye, I A; Wu, S.-S.; Chibaatar, E; Yu, D.-F.; Le, K; Cao, X.-J.; Guo, Y.-J. | 2021 | Neural Plasticity | Search noise |
| **Behavioral health and adult milestones in young adults with perinatal HIV infection or exposure** | Abrams, E J; Mellins, C A; Bucek, A; Dolezal, C; Raymond, J; Wiznia, A; Jurgrau, A; Bamji, M; Leu, C.-S.; Ng, Y K W | 2018 | Pediatrics | Search noise |
| **Deep sedation technique for dental rehabilitation of a patient with Klippel-Feil syndrome** | Abukabbos, H; Mahla, M; Adewumi, A O | 2017 | Journal of Dentistry for Children | Different type of study |
| **Hypnodontics as an alternative to relieve anxiety in children: A systematic review** | Achmad, H; Djais, A I; Rieuwpassa, I E; Riyanti, E; Primarti, R S; Vionita, V | 2021 | Systematic Reviews in Pharmacy | Different type of study |
| **A survey of members of the American Academy of Pediatric Dentistry on their use of behavior management techniques** | Adair, S M; Waller, J L; Schafer, T E; Rockman, R A | 2004 | Pediatric Dentistry | Different outcome |
| **A preliminary study on oxygen saturation levels of patients during periodontal surgery with and without oral conscious sedation using diazepam** | Aeschliman, S D; Blue, M S; Williams, K B; Cobb, C M; MacNeill, S R | 2003 | Journal of Periodontology | Different sample |
| **Imipramine versus placebo for multiple functional somatic syndromes (STreSS-3): a double-blind, randomised study** | Agger, J L; Schroder, A; Gormsen, L K; Jensen, J S; Jensen, T S; Fink, P K | 2017 | The Lancet Psychiatry | Search noise |
| **The prevalence of emergence delirium and its associated factors among children at a postoperative unit: A retrospective cohort at a Middle Eastern hospital** | Aldakhil, S; Salam, M; Albelali, A; Alkanhal, R; Alnemer, M; Alatassi, A | 2020 | Saudi Journal of Anaesthesia | Search noise |
| **Introduction: Methods, commentary, and summary** | Allen, M H; Currier, G W; Carpenter, D; Ross, R W; Docherty, J P | 2005 | Journal of Psychiatric Practice | Search noise |
| **Successful anesthetic and airway management in Coffin-Siris syndrome with congenital heart disease: Case report** | Altun, D; Demir, G; Ayhan, A; Türköz, A | 2016 | Egyptian Journal of Anaesthesia | Search noise |
| **Guideline on appropriate use of nitrous oxide for pediatric dental patients.** | American Academy on Pediatric Dentistry Council on Clinical Affairs | **2008** | **Pediatric dentistry** | **Different type of study** |
| **Fresh approaches to antidepressant drug discovery** | Anacker, C | 2014 | Expert Opinion on Drug Discovery | Search noise |
| **General anesthesia for the provision of dental treatment to adults with developmental disability.** | Ananthanarayan, C; Sigal, M; Godlewski, W | 1998 | Anesthesia progress | Different type of study |
| **Efficacy of premedication with melatonin versus oral midazolam in pediartic patients** | Ansari, G. | 2016 | Iranian Registry of Clinical Trials | Registration protocol |
| **Oral Midazolam Vs Oral Promethazine Premedication Effect on Dental Sedation** | Ansari, G. | 2017 | Iranian Registry of Clinical Trials | Registration protocol |
| **Risperidone treatment for schizotypal personality disorder** | Anwunah, I J; Mitropoulou, V; Bushnoe, L; Siever, L J | 2003 | 51st Institute on Psychiatric Services; 1999 October 25th-November 2nd; New Orleans, LA, USA | Search noise |
| **MDMA treatment paired with a trauma-cue promotes adaptive stress responses in a translational model of PTSD in rats** | Arluk, S; Matar, M A; Carmi, L; Arbel, O; Zohar, J; Todder, D; Cohen, H | 2022 | Translational Psychiatry | Search noise |
| **Pharmaco-therapeutic evaluation of medical treatments of adult autistics and multi-handicapped patients in a public mental health unit** | Armand-Branger, S; Poisson, N; Gaudoneix-Taïeb, M; Ramos, O | 2009 | Encephale | Search noise |
| **Emergency medicine, psychiatry, and the law** | Armitage, D T; Townsend, G M | 1993 | Emergency Medicine Clinics of North America | Search noise |
| **(S)-5-(2′-Fluorophenyl)- N, N-dimethyl-1,2,3,4-tetrahydronaphthalen-2-amine, a Serotonin Receptor Modulator, Possesses Anticonvulsant, Prosocial, and Anxiolytic-like Properties in an Fmr1 Knockout Mouse Model of Fragile X Syndrome and Autism Spectrum Diso** | Armstrong, J L; Casey, A B; Saraf, T S; Mukherjee, M; Booth, R G; Canal, C E | 2020 | ACS Pharmacology and Translational Science | Search noise |
| **Pediatric tooth extractions under sedoanalgesia** | Arpaci, A H; Isik, B | 2016 | Pakistan Journal of Medical Sciences | Different outcome |
| **Tooth extraction in a patient with the long QT syndrome** | Asahi, Y; Niwa, H; Shibutani, T; Hori, T; Takagi, J; Ichibayashi, Y; Matsuura, H | 1995 | Journal of Japanese Dental Society of Anesthesiology | Different type of study |
| **Local Anesthesia for Prostate Biopsy** | Ashley, R.A. | 2007 | ClinicalTrials.gov | Registration protocol |
| **Effects of oral premedication on cognitive status of elderly patients undergoing cardiac catheterization** | Ashraf, J M; Schweiger, M; Vallurupalli, N; Bellantonio, S; Cook, J R | 2015 | Journal of geriatric cardiology | Different sample |
| **ABC of mental health: Addiction and dependence—II: Alcohol** | Ashworth, M; Gerada, C | 1997 | BMJ | Search noise |
| **The use of conscious sedation versus general anesthesia in modern dentistry: rising ethical dilemmas** | Ayalon, S; Gozal, Y; Kaufman, E | 2004 | Refuat ha-peh eha-shinayim (1993) | Different type of study |
| **Observational cohort study of the triggers, diagnoses and outcomes of the medical emergency team (MET) response in adult psychiatry inpatients colocated with acute medical services in Australia** | Azraai, M; Pham, J H; Looi, W F; Wirth, D; Ng, A S L; Babu, U; Saluja, B; Lim, A K H | 2021 | BMJ Open | Search noise |
| **The dental role in smoking cessation advice for patients with mental and behavioural disorders - Time for improvement?** | Baker, R A | 2005 | British Dental Journal | Search noise |
| **Factors associated with disability in a sample of adults with arthritis** | Baruth, M; Wilcox, S; Schoffman, D E; Becofsky, K | 2013 | Disability and health journal | Search noise |
| **Orthodontic treatment for the special needs child** | Becker, A; Chaushu, S; Shapira, J | 2004 | Seminars in Orthodontics | Different type of study |
| **Repeated onabotulinum neurotoxin A injections for drooling in children with neurodisability** | Bekkers, S; Leow, T Y S; Van Hulst, K; Orriëns, L B; Scheffer, A R T; Van Den Hoogen, F J A | 2021 | Developmental Medicine and Child Neurology | Search noise |
| **Delivery of dental care to persons with disabilities and other special populations** | Bennett, C R | 1999 | Work | Different type of study |
| **Enteral sedation: safety, efficacy, and controversy.** | Berthold, C | 2007 | Compendium of continuing education in dentistry (Jamesburg, N.J. : 1995) | Different type of study |
| **Pathological gambling: Relationship to obesity, self-reported chronic medical conditions, poor lifestyle choices, and impaired quality of life** | Black, D W; Shaw, M; McCormick, B; Allen, J | 2013 | Comprehensive Psychiatry | Search noise |
| **Multimodal Rehabilitation Program to Bladder Cancer Patients** | Borre, M. | 2011 | ClinicalTrials.gov | Registration protocol |
| **Treatment strategies for non-cooperative patients** | Bouvy-Berends, E C | 1991 | Nederlands Tijdschrift voor Tandheelkunde | Different type of study |
| **Anesthesiological support for special dental care** | Bouvy-Berends, E C; Makkes, P C | 1990 | Nederlands Tijdschrift voor Tandheelkunde | Different type of study |
| **Sedation or General Anaesthetic for Special Care Patients?** | Boyle, C A; Lane, H | 2020 | Primary dental journal | Different type of study |
| **Oral midazolam for adults with learning disabilities.** | Boyle, C A; Manley, M C; Fleming, G J | 2000 | Dental update | Different type of study |
| **Nitrous Oxide for Analgesia During Colonoscopy** | Bretthauer, M. | 2006 | ClinicalTrials.gov | Registration protocol |
| **A 7-week, randomized, double-blind trial of olanzapine/fluoxetine combination versus lamotrigine in the treatment of bipolar I depression** | Brown, E B; McElroy, S L; Keck Jr., P E; Deldar, A; Adams, D H; Tohen, M; Williamson, D J | 2006 | Journal of Clinical Psychiatry | Search noise |
| **General medicine and surgery for dental practitioners. Part 5 - Psychiatry** | Brown, S; Greenwood, M; Meechan, J G | 2010 | British Dental Journal | Different type of study |
| **Artifactual pseudo-cheilitis: A case series of an underreported condition** | Burns, A; Marchitto, M C; Jhaveri, M; Kang, J; Rozati, S | 2021 | JAAD Case Reports | Search noise |
| **Correlation between blood and oral fluid psychoactive drug concentrations and cognitive impairment in driving under the influence of drugs** | Busardò, F P; Pichini, S; Pellegrini, M; Montana, A; Faro, A F L; Zaami, S; Graziano, S | 2018 | Current Neuropharmacology | Search noise |
| **Prescription Benzodiazepine Use in Privately Insured U.S. Children and Adolescents** | Bushnell, G A; Crystal, S; Olfson, M | 2019 | American Journal of Preventive Medicine | Search noise |
| **Coping with cancer chemotherapy** | Butow, P; Ellis, P; Cox, K | 1998 | Modern Medicine of Australia | Search noise |
| **Antistress properties of antidepressant drugs and their clinical implications** | Calabrese, F; Molteni, R; Riva, M A | 2011 | Pharmacology and Therapeutics | Search noise |
| **Riese v. St. Mary's Hospital and Medical Center.** | California. Court of Appeal, First District, Division 2 | **1988** | **West"s California reporter** | **Different type of study** |
| **Pharmaceutical Opioid Use and Dependence among People Living with Chronic Pain: Associations Observed within the Pain and Opioids in Treatment (POINT) Cohort** | Campbell, G; Nielsen, S; Larance, B; Bruno, R; Mattick, R; Hall, W; Lintzeris, N; Cohen, M; Smith, K; Degenhardt, L | 2015 | Pain Medicine (United States) | Search noise |
| **Standing Sedation and Iocoregional Analgesia in Equine Dental Surgery** | Campoy, L; Sedgwick, S R | 2020 | Veterinary Clinics of North America - Equine Practice | Search noise |
| **Low dose oral ketamine treatment in chronic suicidality: An open-label pilot study** | Can, A T; Hermens, D F; Dutton, M; Gallay, C C; Jensen, E; Jones, M; Scherman, J; Beaudequin, D A; Yang, C; Schwenn, P E; Lagopoulos, J | 2021 | Translational Psychiatry | Search noise |
| **Immediate-release methylphenidate for attention deficit hyperactivity disorder (ADHD) in adults** | Cândido, R C F; Menezes de Padua, C A; Golder, S; Junqueira, D R | 2021 | Cochrane Database of Systematic Reviews | Search noise |
| **Curcumin treatment attenuates alcohol-induced alterations in a mouse model of foetal alcohol spectrum disorders** | Cantacorps, L; Montagud-Romero, S; Valverde, O | 2020 | Progress in Neuro-Psychopharmacology and Biological Psychiatry | Search noise |
| **Conscious sedation with nitrouse oxide-oxigen in dentistry** | Carbone, M; Manno, E | 2012 | Italian Oral Surgery | Different type of study |
| **Endotrol-tracheal Tube Assisted Endotracheal Intubation During Video Laryngoscopy** | Cattano, D | 2010 | ClinicalTrials.gov | Registration protocol |
| **Predictive Indicators For Managing Dental Care In Patients With Different Abilities** | Cava-Arangoitia, O C J; Guevara-Canales, J O; Morales-Vadillo, R; Cava-Vergiú, C E; Uribe, M R; Ortega, L M | 2020 | Cumhuriyet Dental Journal | Different outcome |
| **Use of mild electric stimulation as a distractor in anxious patients** | Cebalo, N; Bašić Kes, V; Verzak, Ž; Karlović, Z; Cebalo, J; Budak, L; Negovetić Vranić, D | 2021 | Acta Stomatologica Croatica | Search noise |
| **Better understanding your patient from a psychological perspective: early identification of problem behaviors affecting the dental office.** | Centore, L; Reisner, L; Pettengill, C A | 2002 | Journal of the California Dental Association | Search noise |
| **Pain and anxiety control in Down syndrome.** | Cetrullo, N; Cocchi, S; Guadagni, M G; Piana, G | 2004 | Minerva stomatologica | Search noise |
| **A review of prolonged post-covid-19 symptoms and their implications on dental management** | Chakraborty, T; Jamal, R F; Battineni, G; Teja, K V; Marto, C M; Spagnuolo, G | 2021 | International Journal of Environmental Research and Public Health | Search noise |
| **Burning mouth syndrome: A review of recent literature topical collection on uncommon headache syndromes** | Charleston IV, L | 2013 | Current Pain and Headache Reports | Search noise |
| **Dysphagia or dysphagias during neuroleptic medication?** | Chaumartin, N; Monville, M; Lachaux, B | 2012 | Encephale | Search noise |
| **Efficacy of botulinum-A for nocturnal bruxism pain and the occurrence of bruxism events: a meta-analysis and systematic review** | Cheng, Y; Yuan, L; Ma, L; Pang, F; Qu, X; Zhang, A | 2022 | British Journal of Oral and Maxillofacial Surgery | Search noise |
| **Few effects of hypnosis on conscious sedation in patients undergoing colonoscopy - A randomized controlled trial** | Cheseaux, N; Forster, A; Frossard, J-L; Dumonceau, J-M; Walder, B | 2011 | European journal of anaesthesiology | Different outcome |
| **Childhood Small Vessel Primary Angiitis of the Central Nervous System: A Treatable Cause of Super-refractory Status Epilepticus** | Chiu, M; Datta, A | 2020 | Journal of Child Neurology | Search noise |
| **Prevalence of dental disorders among people with mental illness: An umbrella review** | Choi, J; Price, J; Ryder, S; Siskind, D; Solmi, M; Kisely, S | 2021 | Australian and New Zealand Journal of Psychiatry | Search noise |
| **The use of aripiprazole in the treatment of mental disorders in children and adolescents** | Cichoń, L; Gabryel, B; Jelonek, I; Krysta, K; Janas-Kozik, M | 2018 | Psychiatria i Psychologia Kliniczna | Search noise |
| **Women with borderline personality disorder** | Clayton, A H | 2004 | Primary Psychiatry | Search noise |
| **Oral health impacts of medications used to treat mental illness** | Cockburn, N; Pradhan, A; Taing, M W; Kisely, S; Ford, P J | 2017 | Journal of Affective Disorders | Search noise |
| **Dental treatment for handicapped patients; Sedation vs general anesthesia and update of dental treatment in patients with different diseases** | Corcuera-Flores, J.-R.; Delgado-Muñoz, J.-M.; Ruiz-Villandiego, J.-C.; Maura-Solivellas, I; Machuca-Portillo, G | 2014 | Medicina Oral, Patologia Oral y Cirugia Bucal | Different outcome |
| **A reply** | Coulthard, P | 2007 | Anaesthesia | Different type of study |
| **Gorham-stout disease successfully treated with sirolimus and zoledronic acid therapy** | Cramer, S L; Wei, S; Merrow, A C; Pressey, J G | 2016 | Journal of Pediatric Hematology/Oncology | Search noise |
| **Treating Phobia With Multivoxel Neuro-reinforcement** | Craske, M. | 2018 | ClinicalTrials.gov | Registration protocol |
| **Lamotrigine for people with borderline personality disorder: A RCT** | Crawford, M J; Sanatinia, R; Barrett, B; Cunningham, G; Dale, O; Ganguli, P; Lawrence-Smith, G; Leeson, V C; Lemonsky, F; Lykomitrou-Matthews, G; Montgomery, A; Morriss, R; Munjiza, J; Paton, C; Skorodzien, I; Singh, V; Tan, W; Tyrer, P; Reilly, J G | 2018 | Health Technology Assessment | Search noise |
| **Mirtazapine: A review of its use in major depression and other psychiatric disorders** | Croom, K F; Perry, C M; Plosker, G L | 2009 | CNS Drugs | Different type of study |
| **Sexual distress in patients with hidradenitis suppurativa: A cross-sectional study** | Cuenca-Barrales, C; Ruiz-Villaverde, R; Molina-Leyva, A | 2019 | Journal of Clinical Medicine | Search noise |
| **Strategies used for the outpatient dental care of people with autism spectrum disorder: An integrative review** | Curi, D S C; Miranda, V.E.V.L.; Barros da Silva, Z; Bem, M.C.D.L.; de Pinho, M D; Zink, A G | 2022 | Research in Autism Spectrum Disorders | Different type of study |
| **Scientific and ethical concerns in clinical trials in Alzheimer's patients: the bridging study** | Cutler, N R; Sramek, J J | 1995 | European Journal of Clinical Pharmacology | Search noise |
| **Training children with autism spectrum disorders to be compliant with an oral assessment** | Cuvo, A J; Godard, A; Huckfeldt, R; Demattei, R | 2010 | Research in Autism Spectrum Disorders | Different outcome |
| **Sedation versus protective stabilization for dental treatment of children with caries and challenging behavior at the dentist (CHOOSE): a study protocol for a non-randomized clinical trial** | da Silva, G S; Anabuki, A A; Viana, K A; Correa-Faria, P; Moterane, M M; Tedesco, T K; Costa, P S; Hosey, M T; Raggio, D P; Costa, L R | 2021 | BMC oral health | Different type of study |
| **Dental anesthesia** | Dall'Oppio, L | 1984 | Dental Cadmos | Abstract not avaible |
| **Levels of stress among general practitioners, students and specialists in pediatric dentistry during dental treatment** | Davidovich, E; Pessov, Y; Baniel, A; Ram, D | 2015 | Journal of Clinical Pediatric Dentistry | Search noise |
| **Restraint and sedation of the dental patient with developmental disabilities** | Davila, J M | 1990 | Special Care in Dentistry | Abstract not avaible |
| **Epilepsy pharmacological treatment and monitoring** | Dawda, Y; Ezewuzie, N | 2010 | Clinical Pharmacist | Search noise |
| **Behavior guidance techniques in Pediatric Dentistry: Attitudes of parents of children with disabilities and without disabilities** | De Castro, A M; De Oliveira, F S; De Paiva Novaes, M S; Araújo Ferreira, D C | 2013 | Special Care in Dentistry | Search noise |
| **Poor Adherence to Oral Psychiatric Medication in Adults with Depression: Psychological Reactance May Have Specific Effects in Depression** | De Las Cuevas, C; Motuca, M; Baptista, T; Villasante-Tezano, A G; Lazary, J; Pogany, L; De Leon, J | 2021 | Neuropsychopharmacologia Hungarica | Search noise |
| **Dental care in autism** | De Moor, R; Martens, L | 1997 | Revue belge de médecine dentaire. Belgisch tijdschrift voor tandheelkunde | Different type of study |
| **Ketamine and other glutamate receptor modulators for depression in adults with unipolar major depressive disorder** | Dean, R L; Hurducas, C; Hawton, K; Spyridi, S; Cowen, P J; Hollingsworth, S; Marquardt, T; Barnes, A; Smith, R; McShane, R; Turner, E H; Cipriani, A | 2021 | Cochrane Database of Systematic Reviews | Search noise |
| **Optimization of Procedural Sedation Protocol Used for Dental Care Delivery in People With Mental Disability** | Declerck, D. | 2013 | ClinicalTrials.gov | Registration protocol |
| **Efficacy of Dexamethasone or Adrenaline in Inferior Alveolar Nerve Block** | Deo, S.P. | 2021 | ClinicalTrials.gov | Registration protocol |
| **Dental care in children with down syndrome: A questionnaire for belgian dentists** | Descamps, I; Fernandez, C; Van Cleynenbreugel, D; Van Hoecke, Y; Marks, L | 2019 | Medicina Oral Patologia Oral y Cirugia Bucal | Different outcome |
| **Treatment of a serious autistic disorder in a child with Naltrexone in an oral suspension form** | Desjardins, S; Doyen, C; Contejean, Y; Kaye, K; Paubel, P | 2009 | Encephale | Search noise |
| **Optimising oral health in frail older people** | Deutsch, A; Jay, E | 2021 | Australian Prescriber | Different sample |
| **Prescription drug benefits and Canada's uninsured** | Dewa, C S; Hoch, J S; Steele, L | 2005 | International Journal of Law and Psychiatry | Search noise |
| **Double-Blind, Randomized, Placebo Controlled Clinical Trial Examining the Efficacy of Steroid Supplementation after TMJ Arthrocentesis** | Diaz, D; Dolwick, M F; Freburg-Hoffmeister, D L; Widmer, C G | 2019 | Journal of oral and maxillofacial surgery | Search noise |
| **Oral sedation.** | Dionne, R | 1998 | Compendium of continuing education in dentistry (Jamesburg, N.J. : 1995) | Different type of study |
| **Hyposalivation and xerostomia in schizophrenic patients on psychotropic medications** | Djordjevic, V; Djokic, G; Domic, D; Zivkovic, N; Jankovic, L; Milicic, B; Djukic Dejanovic, S | 2016 | European neuropsychopharmacology | Conference poster |
| **Pharmaceutical drug misuse in Australia** | Dobbin, M | 2014 | Australian Prescriber | Search noise |
| **Weight Loss and Lowering Androgens Predict Improvements in Health-Related Quality of Life in Women With PCOS** | Dokras, A; Sarwer, D B; Allison, K C; Milman, L; Kris-Etherton, P M; Kunselman, A R; Stetter, C M; Williams, N I; Gnatuk, C L; Estes, S J; al., et | 2016 | Journal of clinical endocrinology and metabolism | Search noise |
| **Homeopathic approach in the treatment of patients with mental disability** | Dolce Filho, R | 2006 | Homeopathy | Search noise |
| **Effect of dimebon on cognition, activities of daily living, behaviour, and global function in patients with mild-to-moderate Alzheimer's disease: a randomised, double-blind, placebo-controlled study** | Doody, R S; Gavrilova, S I; Sano, M; Thomas, R G; Aisen, P S; Bachurin, S O; Seely, L; Hung, D | 2008 | The Lancet | Search noise |
| **Is intravenous conscious sedation for surgical orthodontics in children a viable alternative to general anaesthesia? - A case review** | Dorman, M L; Wilson, K; Stone, K; Stassen, L F A | 2007 | British Dental Journal | Different sample |
| **Burning mouth syndrome** | Drage, L A; Rogers III, R S | 2003 | Dermatologic Clinics | Search noise |
| **Characterizing and improving HIV and hepatitis knowledge among primary prescription opioid abusers** | Dunn, K E; Saulsgiver, K A; Patrick, M E; Heil, S H; Higgins, S T; Sigmon, S C | 2013 | Drug and alcohol dependence | Search noise |
| **Are special care dentistry services prepared for a global disruption in healthcare? A call for a wider promotion of dental conscious sedation training** | Dziedzic, A; Tanasiewicz, M; Abed, H; Dickinson, C; Picciani, B | 2020 | Healthcare (Switzerland) | Different type of study |
| **Advances in medicinal plants with effects on anxiety behavior associated to mental and health conditions** | Echeverria, V; Aliev, G; Foitzick, M; Avila-Rodriguez, M; Barreto, G E | 2017 | Current Medicinal Chemistry | Search noise |
| **Parental acceptance of pediatric behavior management techniques: A comparative study** | Elango, I; Baweja, D; Shivaprakash, P | 2012 | Journal of Indian Society of Pedodontics and Preventive Dentistry | Search noise |
| **A new clinical protocol for the pharmacological management of acute behavioural disturbance** | Emmerson, B; Moudgil, V; Woodbridge, A; Burrows, D; Kingswell, B; Kubler, P; McMahon, K | 2011 | Australasian Psychiatry | Search noise |
| **Evaluation and comparison the effectiveness of oral melatonin and midazolam as premedication on sedation and post operative complications of candidate children for dental treatment under general anesthesia with contral group** | Faghihian, R. | 2016 | Iranian Registry of Clinical Trials | Registration protocol |
| **Effectiveness of using external cold and vibration on local anesthesia pain** | Faghihian, R. | 2021 | Iranian Registry of Clinical Trials | Registration protocol |
| **Effect of cold and external vibration in local anesthesia** | Faghihian, R. | 2021 | Iranian Registry of Clinical Trials | Registration protocol |
| **Experience with sedation and restraint during dental treatment in Romania.** | Fanning, B | 1995 | Journal of the Irish Dental Association | Different type of study |
| **The World Federation of ADHD International Consensus Statement: 208 Evidence-based conclusions about the disorder** | Faraone, S V; Banaschewski, T; Coghill, D; Zheng, Y; Biederman, J; Bellgrove, M A; Newcorn, J H; Gignac, M; Al Saud, N M; Manor, I; Rohde, L A; Yang, L; Cortese, S; Almagor, D; Stein, M A; Albatti, T H; Aljoudi, H F; Alqahtani, M M J; Asherson, P; Atwoli, L; Bölte, S; Buitelaar, J K; Crunelle, C L; Daley, D; Dalsgaard, S; Döpfner, M; Espinet, S; Fitzgerald, M; Franke, B; Gerlach, M; Haavik, J; Hartman, C A; Hartung, C M; Hinshaw, S P; Hoekstra, P J; Hollis, C; Kollins, S H; Sandra Kooij, J J; Kuntsi, J; Larsson, H; Li, T; Liu, J; Merzon, E; Mattingly, G; Mattos, P; McCarthy, S; Mikami, A Y; Molina, B S G; Nigg, J T; Purper-Ouakil, D; Omigbodun, O O; Polanczyk, G V; Pollak, Y; Poulton, A S; Rajkumar, R P; Reding, A; Reif, A; Rubia, K; Rucklidge, J; Romanos, M; Ramos-Quiroga, J A; Schellekens, A; Scheres, A; Schoeman, R; Schweitzer, J B; Shah, H; Solanto, M V; Sonuga-Barke, E; Soutullo, C; Steinhausen, H.-C.; Swanson, J M; Thapar, A; Tripp, G; van de Glind, G; Brink, W V D; Van der Oord, S; Venter, A; Vitiello, B; Walitza, S; Wang, Y | 2021 | Neuroscience and Biobehavioral Reviews | Different type of study |
| **Levetiracetam for managing neurologic and psychiatric disorders** | Farooq, M U; Bhatt, A; Majid, A; Gupta, R; Khasnis, A; Kassab, M Y | 2009 | American Journal of Health-System Pharmacy | Search noise |
| **A prospective hospital based study to monitor the adverse drug reactions of antidepressant drugs in psychiatric department of a tertiary care hospital.** | Farooq, S; Farhat, S; Koul, R K; Rather, Y H | 2018 | JK Practitioner | Search noise |
| **Management of an oral ingestion of transdermal fentanyl patches: A case report and literature review** | Faust, A C; Terpolilli, R; Hughes, D W | 2011 | Case Reports in Medicine | Search noise |
| **Antipsychotics, mood stabilisers, and risk of violent crime** | Fazel, S; Zetterqvist, J; Larsson, H; Långström, N; Lichtenstein, P | 2014 | The Lancet | Search noise |
| **Erectile dysfunction: Management update** | Fazio, L; Brock, G | 2004 | CMAJ | Search noise |
| **Preparing for Puberty in Girls With Special Needs: A Cohort Study of Caregiver Concerns and Patient Outcomes** | Fei, Y F; Ernst, S D; Dendrinos, M L; Quint, E H | 2021 | Journal of Pediatric and Adolescent Gynecology | Search noise |
| **Methylphenidate (ritalin) hydrochloride parenteral solution: Preliminary report** | Ferguson, J T; Linn, F V Z; Sheets Jr., J A; Nickels, M M | 1956 | Journal of the American Medical Association | Different type of study |
| **Is hydrogen water intervention and cognitive-behavioral group therapy effective in women suffering from panic attacks?** | Fernández Serrano, A.B. | 2021 | ISRCTN Registry | Registration protocol |
| **Natural history of insomnia symptoms in the transition from childhood to adolescence: Population rates, health disparities, and risk factors** | Fernandez-Mendoza, J; Bourchtein, E; Calhoun, S; Puzino, K; Snyder, C K; He, F; Vgontzas, A N; Liao, D; Bixler, E | 2021 | Sleep | Search noise |
| **Psychiatric medicine and the care of non-acute hospitalized patients with mental retardation and mental illness or severe behavior disorders** | Fleisher, M H | 2003 | Mental Health Aspects of Developmental Disabilities | Search noise |
| **Continuous versus intermittent treatment with citalopram in premenstrual dysphoric disorder** | Flores Ramos, M; Ontiveros Uribe, M; Cortes Sotres, J | 2003 | Salud mental (Mexico City, Mexico) | Search noise |
| **A review of the pharmacological approach to the management of dental anxiety in children** | Folayan, M O; Faponle, A; Lamikanra, A | 2002 | International Journal of Paediatric Dentistry | Different type of study |
| **Chloral hydrate as a sedating agent for neurodiagnostic procedures in children** | Fong, C Y; Lim, W K; Li, L; Lai, N M | 2021 | Cochrane Database of Systematic Reviews | Different type of study |
| **The CINP guidelines on the definition and evidence-based interventions for treatment-resistant bipolar disorder** | Fountoulakis, K N; Yatham, L N; Grunze, H; Vieta, E; Young, A H; Blier, P; Tohen, M; Kasper, S; Moeller, H J | 2020 | International Journal of Neuropsychopharmacology | Different type of study |
| **The handicapped child. A prelude to care** | Fox, L A | 1974 | Dental Clinics of North America | Different type of study |
| **Oral management of Steinert's disease and role of anxiolysis** | Franco, R; Miranda, M; Di Renzo, L; Barlattani, A; De Lorenzo, A; Bollero, P | 2018 | Journal of Contemporary Dental Practice | Search noise |
| **Dental management of child and adolescent patients with schizophrenia.** | Friedlander, A H; Friedlander, I K; Eth, S; Freymiller, E G | 1993 | ASDC journal of dentistry for children | Different type of study |
| **The adult suicide-prone patient: A review of the medical literature and implications for oral and maxillofacial surgeons** | Friedlander, A H; Rosenbluth, S C; Rubin, R T | 2012 | Journal of Oral and Maxillofacial Surgery | Search noise |
| **Social stress in tree shrews as an animal model of depression: An example of a behavioral model of a CNS disorder** | Fuchs, E | 2005 | CNS Spectrums | Search noise |
| **Fitting complete dentures after multiple tooth extraction in a patient with severe dementia** | Fujisawa, T; Yokoyama, A; Muramatsu, M; Kimura, Y; Kurozumi, A; Kobayashi, I; Sano, H; Totsuka, Y; Fukushima, K | 2007 | Special Care in Dentistry | Different type of study |
| **Dental treatment for the handicapped by a combination of public community, dental association and university hospital--a system of Suginoki Dental Clinic** | Fukayama, H; Matsumoto, M; Kohase, H; Sato, O; Akiyama, H; Hirai, Y; Yazawa, M; Umino, M | 2000 | Kōkūbyō Gakkai zasshi. The Journal of the Stomatological Society, Japan | Different type of study |
| **Using intravenous sedation to manage adults with neurological impairment** | Galli, M T | 1999 | Special Care in Dentistry | Different type of study |
| **ECT With Ketamine Anesthesia vs High Intensity Ketamine With ECT Rescue for Treatment-Resistant Depression** | Gamble, J. | 2017 | ClinicalTrials.gov | Registration protocol |
| **Deep sedation and GA** | Ganzberg, S I | 2015 | Oral Sedation for Dental Procedures in Children | Different type of study |
| **Long-Acting Injectable Antipsychotics: Analysis of Prescription Patterns and Patient Characteristics in Mental Health from a Spanish Real-World Study** | García-Carmona, J A; Simal-Aguado, J; Campos-Navarro, M P; Valdivia-Muñoz, F; Galindo-Tovar, A | 2020 | Clinical Drug Investigation | Search noise |
| **A comparison of midazolam and midazolam with remifentanil for patient-controlled sedation during operations on third molars** | Garip, H; Gürkan, Y; Toker, K; Göker, K | 2007 | British Journal of Oral and Maxillofacial Surgery | Different sample |
| **Assessment and management of agitation in psychiatry: Expert consensus** | Garriga, M; Pacchiarotti, I; Kasper, S; Zeller, S L; Allen, M H; Vázquez, G; Baldacąra, L; San, L; McAllister-Williams, R H; Fountoulakis, K N; Courtet, P; Naber, D; Chan, E W; Fagiolini, A; Möller, H J; Grunze, H; Llorca, P M; Jaffe, R L; Yatham, L N; Hidalgo-Mazzei, D; Passamar, M; Messer, T; Bernardo, M; Vieta, E | 2016 | World Journal of Biological Psychiatry | Search noise |
| **Awareness about management of pain and anxiety during dental treatments among dental students** | Gayathri, P S; Krithika, C; Arun Kumar, T M; Selvarathi, K; Christy Jospeh Samuel, K; Manju, J | 2021 | Indian Journal of Forensic Medicine and Toxicology | Different outcome |
| **The influence of the covid-19 pandemic on the stress levels and occurrence of stomatoghnatic system disorders (Ssds) among physiotherapy students in poland** | Gębska, M; Kołodziej, Ł; Dalewski, B; Pałka, Ł; Sobolewska, E | 2021 | Journal of Clinical Medicine | Search noise |
| **Safety and Efficacy of Escitalopram in the Treatment of Premature Ejaculation** | Ghanem, M.H. | 2008 | ClinicalTrials.gov | Registration protocol |
| **General anesthesia protocol for the dental patient: Emphasis for older adults** | Ghezzi, E M | 2000 | Special Care in Dentistry | Search noise |
| **Prevalence of psychiatric disorders in a group of adult patients seeking general dental care** | Giglio, J A; Laskin, D M | 2010 | Quintessence International | Search noise |
| **A review of guidelines for sedation, anesthesia, and alternative interventions for people with special needs** | Glassman, P | 2009 | Special Care in Dentistry | Different type of study |
| **Special care dentistry association consensus statement on sedation, anesthesia, and alternative techniques for people with special needs** | Glassman, P; Caputo, A; Dougherty, N; Lyons, R; Messieha, Z; Miller, C; Peltier, B; Romer, M | 2009 | Special Care in Dentistry | Different type of study |
| **Social supports and prevention strategies as adjuncts and alternatives to sedation and anesthesia for people with special needs** | Glassman, P; Miller, C | 2009 | Special Care in Dentistry | Different type of study |
| **Transplantation of mesenchymal stem cells causes long-term alleviation of schizophrenia-like behaviour coupled with increased neurogenesis** | Gobshtis, N; Tfilin, M; Fraifeld, V E; Turgeman, G | 2021 | Molecular Psychiatry | Search noise |
| **Reproductive health in an inpatient psychiatric unit: A retrospective chart review** | Goldstein, N; Davis, C; Saliba, Z | 2021 | Human Psychopharmacology | Search noise |
| **The Impact of the Motivational Interview on the behaviors and conditions of individuals with disorders for the use of psychotropic substances - a randomized clinical trial** | Gomes Lopes, A. | 2018 | Registro Brasilero de Ensaios Clinicos | Registration protocol |
| **Dexamethasone and Respiratory Function After Mastectomy** | González-Ojeda, A. | 2014 | ClinicalTrials.gov | Registration protocol |
| **Dental fear and anxiety as a barrier to accessing oral health care among patients with special health care needs** | Gordon, S M | 1998 | Special Care in Dentistry | Different outcome |
| **Dental anxiety and behavioural problems: What is their influence on the treatment plan?** | Goumans, C; Veerkamp, J S J; Aartman, I H A | 2004 | European Journal of Paediatric Dentistry | Different type of study |
| **Mental illness and the Quality Use of Medicines** | Gowan, J; Roller, L | 2003 | Australian Journal of Pharmacy | Search noise |
| **A Clinical Study to Evaluate the Effectiveness and Safety of Zolpidem Tartrate Combined with Cognitive Behavioral Therapy Versus Cognitive Behavioral Therapy Alone in Patients with Insomnia Associated with Comorbid Anxiety** | Gowda, M. | 2020 | Clinical Trial Registry-India | Registration protocol |
| **Prevalence and correlates of common mental disorders among dental students in Brazil** | Graner, K M; De Moraes, A B A; Torres, A R; Lima, M C P; Rolim, G S; De Abreu Ramos-Cerqueira, A T | 2018 | PLoS ONE | Search noise |
| **Sedation--does it decrease the stress of general practice?** | Gustafson, K | 1995 | SAAD digest | Different outcome |
| **Onychophagia: A nail-biting conundrum for physicians** | Halteh, P; Scher, R K; Lipner, S R | 2017 | Journal of Dermatological Treatment | Search noise |
| **Methamphetamine abuse and dentistry** | Hamamoto, D T; Rhodus, N L | 2009 | Oral Diseases | Search noise |
| **Application Effect of Computer-Assisted Local Anesthesia in Patient Operation** | Hao, Y; Zhang, Z; Meng, Y | 2021 | Contrast media & molecular imaging | Search noise |
| **Electronic cigarettes for smoking cessation** | Hartmann-Boyce, J; McRobbie, H; Lindson, N; Bullen, C; Begh, R; Theodoulou, A; Notley, C; Rigotti, N A; Turner, T; Butler, A R; Fanshawe, T R; Hajek, P | 2020 | Cochrane Database of Systematic Reviews | Search noise |
| **Interventions for preventing weight gain after smoking cessation** | Hartmann-Boyce, J; Theodoulou, A; Farley, A; Hajek, P; Lycett, D; Jones, L L; Kudlek, L; Heath, L; Hajizadeh, A; Schenkels, M; Aveyard, P | 2021 | Cochrane Database of Systematic Reviews | Search noise |
| **Intravenous sedation with dexmedetomidine for dental outpatients** | Hashimoto, K; Sugioka, S; Kato, Y; Kotani, J | 2005 | Journal of Japanese Dental Society of Anesthesiology | Different type of study |
| **Lamotrigine in the maintenance treatment of bipolar disorder** | Hashimoto, Y; Kotake, K; Watanabe, N; Fujiwara, T; Sakamoto, S | 2021 | Cochrane Database of Systematic Reviews | Search noise |
| **Targeting ligand-operated chaperone sigma-1 receptors in the treatment of neuropsychiatric disorders** | Hayashi, T; Tsai, S.-Y.; Mori, T; Fujimoto, M; Su, T.-P. | 2011 | Expert Opinion on Therapeutic Targets | Search noise |
| **Self‐assessment of dental health among Danish noninstitutionalized psychiatric patients** | Hede, B; Petersen, P E | 1992 | Special Care in Dentistry | Search noise |
| **A national study of prescribed drugs in institutions and community residential facilities for mentally retarded people** | Hill, B K; Balow, E A; Bruininks, R H | 1985 | Psychopharmacology Bulletin | Search noise |
| **Management strategies for adult patients with dental anxiety in the dental clinic: A Systematic Review** | Hoffmann, B; Erwood, K; Ncomanzi, S; Fischer, V; O'Brien, D; Lee, A | 2022 | Australian dental journal | Different type of study |
| **A digital fabrication of dental prosthesis for preventing self-injurious behavior related to autism spectrum disorder: A case report** | Hong, S.-J.; Chae, Y K; Lee, C; Choi, S C; Nam, O H | 2021 | International Journal of Environmental Research and Public Health | Different type of study |
| **Olanzapine orally disintegrating tablets (Zyprexa ZydisR) rapidly improve excitement components in the acute phase of first-episode schizophrenic patients: An open-label prospective study** | Hori, H; Ueda, N; Yoshimura, R; Yamamoto, H; Wani, K; Etoh, Y; Haraga, K; Kitahara, J; Nakamura, J | 2009 | World Journal of Biological Psychiatry | Search noise |
| **The Effect Of Jaw Relaxation On Anxiety and Sleep Quality of Patients with Myocardial Infarction** | Hoseyni, M. | 2016 | Iranian Registry of Clinical Trials | Registration protocol |
| **Hospital-based dental care for persons with disabilities: A study of patient selection criteria** | Hulland, S; Sigal, M J | 2000 | Special Care in Dentistry | Search noise |
| **Knowledge and practice of behavioral management principles among dentists treating adults with learning disabilities** | Humza Bin Saeed, M; Daly, B; Newton, J T | 2012 | Special Care in Dentistry | Different outcome |
| **Patient attitudes towards surgically implantable, long-term delivery of psychiatric medicine** | Irani, F; Dankert, M; Brensinger, C; Bilker, W B; Nair, S R; Kohler, C G; Kanes, S J; Turetsky, B I; Moberg, P J; Ragland, J D; Gur, R C; Gur, R E; Siegel, S J | 2004 | Neuropsychopharmacology | Search noise |
| **Anesthetic management for a patient with mental retardation and unexamined complex congenital heart disease** | Iribe, G; Yoshimine, K; Takehara, A; Masuda, M; Omae, T; Kamihashi, M | 2000 | Japanese Journal of Anesthesiology | Search noise |
| **Collaboration of perioperative management in an adult patient with 22 q 11.2 deletion syndrome: A case report** | Ito, M; Tokura, T; Miyauchi, T; Sato, A; Kimura, H; Tsuchihashi, H; Katayama, Y | 2022 | Clinical Case Reports | Different type of study |
| **A Survey for Intravenous Sedation in Mentally Retarded and Physically Disabled - Results of Questionnaire from Four University Dental Hospitals in Kantoh Area** | Itoh, K; Sunada, K; Fujisaki, R; Miura, A; Shinohara, K; Yamashiro, M; Sumitomo, M; Furuya, H; Itoh, H | 2004 | Journal of Japanese Dental Society of Anesthesiology | Different sample |
| **Psychotropic drugs in pregnancy and lactation** | Jain, A E; Lacy, T | 2005 | Journal of Psychiatric Practice | Search noise |
| **Historical perspective on antipsychotic long-acting injections** | Johnson, D A W | 2009 | British Journal of Psychiatry | Search noise |
| **New information for our special care of patients with dementia.** | Jones, J A | 2000 | Special care in dentistry : official publication of the American Association of Hospital Dentists, the Academy of Dentistry for the Handicapped, and the American Society for Geriatric Dentistry | Abstract not avaible |
| **Considerations in the dental care of the mentally ill** | Joris, R | 2005 | Schweizer Monatsschrift für Zahnmedizin = Revue mensuelle suisse d'odonto-stomatologie = Rivista mensile svizzera di odontologia e stomatologia / SSO | Abstract not avaible |
| **Why would I smile?** | Joshi, K G | 2017 | BMJ (Online) | Abstract not avaible |
| **DL-/PO-phosphatidylcholine restores restraint stress-induced depression-related behaviors and spatial memory impairment** | Kanno, T; Jin, Y; Nishizaki, T | 2014 | Behavioural Pharmacology | Search noise |
| **An evidence-based review of insomnia treatment in early recovery** | Kaplan, K A; McQuaid, J; Primich, C; Rosenlicht, N | 2014 | Journal of Addiction Medicine | Search noise |
| **Treatment challenges in the acute hospital setting** | Kasper, S | 2005 | World Journal of Biological Psychiatry | Abstract not avaible |
| **Alternative cognitive therapy for emotional instability (pathologic laughing and crying)** | Kasprisin, A | 2004 | Physical Medicine and Rehabilitation Clinics of North America | Search noise |
| **Iron deficiency: A diagnostic and therapeutic perspective in psychiatry** | Kassir, A | 2017 | Encephale | Search noise |
| **Sleep Bruxism: A Sleep-Related Movement Disorder** | Kato, T; Lavigne, G J | 2010 | Sleep Medicine Clinics | Search noise |
| **Comparison between intranasal and intravenous midazolam sedation (with or without patient control) in a dental phobia clinic** | Kaufman, E; Davidson, E; Sheinkman, Z; Magora, F | 1994 | Journal of oral and maxillofacial surgery | Different sample |
| **Transient suppression of involuntary movements in cerebral palsy patients during dental treatment** | Kaufman, E; Meyer, S; Wolnerman, J S; Gilai, A N | 1991 | Anesthesia Progress | Different outcome |
| **A study of local anesthesia injection effects on recovery condition for children following dentistry under general anesthesia** | Kaviani, N. | 2011 | Iranian Registry of Clinical Trials | Registration protocol |
| **Continuous infusion propofol general anesthesia for dental treatment in patients with progressive muscular dystrophy.** | Kawaai, H; Tanaka, K; Yamazaki, S | 2005 | Anesthesia progress | Different sample |
| **CNS adverse events associated with antiepileptic drugs** | Kennedy, G M; Lhatoo, S D | 2008 | CNS Drugs | Search noise |
| **IBS07.01 Enhanced Recovery for Thoracic Surgery** | Kerr, A | 2019 | Journal of thoracic oncology | Search noise |
| **A multicentre, randomized, naturalistic, open-label study between aripiprazole and standard of care in the management of community-treated schizophrenic patients Schizophrenia Trial of Aripiprazole: (STAR) study** | Kerwin, R; Millet, B; Herman, E; Banki, C M; Lublin, H; Pans, M; Hanssens, L; L'Italien, G; McQuade, R D; Beuzen, J N | 2007 | European psychiatry | Search noise |
| **Nonpharmacological interventions in patients with cognitive impairment: A comparison of residential and nursing homes in Poland** | Kijowska, V; Barańska, I; Stodolska, A; Szczerbińska, K | 2021 | Polish Archives of Internal Medicine | Search noise |
| **The dental care of the psychiatric patient.** | King, K C | 1998 | The New Zealand dental journal | Different type of study |
| **Evaluation and treatment of female sexual disorders** | Kingsberg, S; Althof, S E | 2009 | International Urogynecology Journal | Search noise |
| **Behavior management issues for pediatric patients.** | Klein, A | 1991 | The Journal of the American Dental Association | Different type of study |
| **Routine drug monitoring of serum concentrations of morphine, morphine-3-glucuronide and morphine-6-glucuronide do not predict clinical observations in cancer patients** | Klepstad, P; Borchgrevink, P C; Dale, O; Zahlsen, K; Aamo, T; Fayers, P; Fougner, B; Kaasa, S | 2003 | Palliative Medicine | Search noise |
| **A survey of specialist paediatric dental services in Sweden: Results from 2003, and trends since 1983** | Klingberg, G; Dahllöf, G; Erlandsson, A.-L.; Grindefjord, M; Hallström-Stalin, U; Koch, G; Lundin, S.-Å. | 2006 | International Journal of Paediatric Dentistry | Different outcome |
| **Biopsychosocial assessment of dental neglect in a pediatric cancer patient from the perspectives of developmental trauma disorder** | Kobayashi, K; Koyama, N; Nagano, Y; Usami, I; Fujimura, K; Heike, T | 2022 | Pediatric Dental Journal | Search noise |
| **The effect of individual consultation on the reduced fear of childbirth among nulliparous women** | Kordi, M. | 2016 | Iranian Registry of Clinical Trials | Registration protocol |
| **The adverse effects of antiepileptic drugs in children** | Kothare, S V; Kaleyias, J | 2007 | Expert Opinion on Drug Safety | Search noise |
| **Slow-release naltrexone implant versus oral naltrexone for improving treatment outcomes in people with HIV who are addicted to opioids: a double-blind, placebo-controlled, randomised trial** | Krupitsky, E; Blokhina, E; Zvartau, E; Verbitskaya, E; Lioznov, D; Yaroslavtseva, T; Palatkin, V; Vetrova, M; Bushara, N; Burakov, A; Masalov, D; Mamontova, O; Langleben, D; Poole, S; Gross, R; Woody, G | 2019 | The Lancet HIV | Search noise |
| **Disappearance of self-aggressive behavior in a brain-injured patient after deep brain stimulation of the hypothalamus: Technical case report** | Kuhn, J; Lenartz, D; Mai, J K; Huff, W; Klosterkoetter, J; Sturm, V | 2008 | Neurosurgery | Search noise |
| **Zuclopenthixol dihydrochloride for schizophrenia** | Kumar, A; Strech, D | 2009 | Cochrane Database of Systematic Reviews | Search noise |
| **Perioperative Enhancement of Cognitive Trajectory (The PROTECT trial)** | L. Evered | 2019 | Australian New Zeland Clinical Trials Registry | Registration protocol |
| **Treatment of sleep disorders in children** | Lam, J C; Mason, T B A | 2007 | Current Treatment Options in Neurology | Search noise |
| **Royal Australian and New Zealand College of Psychiatrists expert consensus statement for the treatment, management and monitoring of the physical health of people with an enduring psychotic illness** | Lambert, T J R; Reavley, N J; Jorm, A F; Oakley Browne, M A | 2017 | Australian and New Zealand Journal of Psychiatry | Search noise |
| **No. 279-Female Sexual Health Consensus Clinical Guidelines** | Lamont, J; Bajzak, K; Bouchard, C; Burnett, M; Byers, S; Cohen, T; Fisher, W; Holzapfel, S; Senikas, V | 2018 | Journal of Obstetrics and Gynaecology Canada | Search noise |
| **Medical, psychological and social features in a large cohort of adults with Prader-Willi syndrome: Experience from a dedicated centre in France** | Laurier, V; Lapeyrade, A; Copet, P; Demeer, G; Silvie, M; Bieth, E; Coupaye, M; Poitou, C; Lorenzini, F; Labrousse, F; Molinas, C; Tauber, M; Thuilleaux, D; Jauregi, J | 2015 | Journal of Intellectual Disability Research | Search noise |
| **Report of the annual conference day. The 44th Annual SAAD Conference Day.** | Leitch, J | 2002 | SAAD digest | Search noise |
| **Metoclopramide as Treatment of Clozapine-induced Hypersalivation** | Lerner, V. | 2014 | ClinicalTrials.gov | Registration protocol |
| **Creatine as a New Therapeutic Strategy in Depression** | Levine, J.; Nemets, B. | 2006 | ClinicalTrials.gov | Registration protocol |
| **Ten years of clinical experience with clozapine about 170 patients** | Levoyer, D; Martinet, J.-P.; Badiche, A; Millet, B | 2004 | Encephale | Search noise |
| **Conscious sedation service for geriatric and special-care dentistry: A health policy brief** | Lim, G X D; Boyle, C A | 2020 | Proceedings of Singapore Healthcare | Different type of study |
| **Perioperative airway management in a child with Treacher Collins syndrome** | Lin, T.-C.; Soo, L.-Y.; Chen, T.-I.; Lu, I.-C.; Hsu, H.-T.; Chu, K.-S.; Yen, M.-K. | 2009 | Acta Anaesthesiologica Taiwanica | Search noise |
| **The GKT diploma in dental sedation - A judgement** | Little, J M; Manley, M C G; Craig, D C | 2004 | British Dental Journal | Different outcome |
| **Anxiety disorders: Dental implications** | Little, J W | 2003 | General Dentistry | Different type of study |
| **Regulation of glutamate transporter 1 via BDNF-TrkB signaling plays a role in the anti-apoptotic and antidepressant effects of ketamine in chronic unpredictable stress model of depression** | Liu, W.-X.; Wang, J; Xie, Z.-M.; Xu, N; Zhang, G.-F.; Jia, M; Zhou, Z.-Q.; Hashimoto, K; Yang, J.-J. | 2016 | Psychopharmacology | Search noise |
| **Behaviour guidance in dental treatment of patients with autism spectrum disorder** | Loo, C Y; Graham, R M; Hughes, C V | 2009 | International Journal of Paediatric Dentistry | Different type of study |
| **New boundaries and dissociation of the mouse hippocampus along the dorsal-ventral axis based on glutamatergic, GABAergic and catecholaminergic receptor densities** | Lothmann, K; Deitersen, J; Zilles, K; Amunts, K; Herold, C | 2021 | Hippocampus | Search noise |
| **The Pro-neurogenic Effects of Cannabidiol and Its Potential Therapeutic Implications in Psychiatric Disorders** | Luján, M Á; Valverde, O | 2020 | Frontiers in Behavioral Neuroscience | Search noise |
| **Understanding basic behavioral support techniques as an alternative to sedation and anesthesia** | Lyons, R A | 2009 | Special Care in Dentistry | Search noise |
| **Perioperative management of a patient with sotos syndrome using visual pedagogy** | M; Nogami, K; Tominaga, S; Katoh, Y; Kubota, T; Taniguchi, S | 2008 | Journal of Japanese Dental Society of Anesthesiology | Different type of study |
| **An examination of psychotropic medication side effects: Does taking a greater number of psychotropic medications from different classes affect presentation of side effects in adults with ID?** | Mahan, S; Holloway, J; Bamburg, J W; Hess, J A; Fodstad, J C; Matson, J L | 2010 | Research in Developmental Disabilities | Search noise |
| **Comparison of efficacy, safety, and cost-effectiveness of montelukast-levocetirizine and montelukast-fexofenadine in patients of allergic rhinitis: a randomized, double-blind clinical trial** | Mahatme, M S; Dakhale, G N; Tadke, K; Hiware, S K; Dudhgaonkar, S D; Wankhede, S | 2016 | Indian journal of pharmacology | Search noise |
| **Intravenous sedation for conservative dentistry for disabled patients** | Malamed, S F; Gottschalk, H W; Mulligan, R; Quinn, C L | 1989 | Anesthesia Progress | Abstract not avaible |
| **Paradoxical reactions to benzodiazepines: Literature review and treatment options** | Mancuso, C E; Tanzi, M G; Gabay, M | 2004 | Pharmacotherapy | Different type of study |
| **Dental treatment for people with challenging behaviour: General anaesthesia or sedation?** | Manley, M C G | 2000 | British Dental Journal | Different type of study |
| **Controlled trial of prescribed heroin in the treatment of opioid addiction** | March, J C; Oviedo-Joekes, E; Perea-Milla, E; Carrasco, F | 2006 | Journal of substance abuse treatment | Search noise |
| **The impact of shared decision-making on the treatment of anxiety and depressive disorders: Systematic review** | Marshall, T; Stellick, C; Abba-Aji, A; Lewanczuk, R; Li, X.-M.; Olson, K; Vohra, S | 2021 | BJPsych Open | Search noise |
| **Periodontal diseases and depression: A pre-clinical in vivo study** | Martínez, M; Martín-Hernández, D; Virto, L; MacDowell, K S; Montero, E; González-Bris, Á; Marín, M J; Ambrosio, N; Herrera, D; Leza, J C; Sanz, M; García-Bueno, B; Figuero, E | 2021 | Journal of Clinical Periodontology | Search noise |
| **Considerations for the use of oral sedation in the institutionalized geriatric patient during dental interventions: A review of the literature** | Matear, D W | 1999 | Special Care in Dentistry | Different type of study |
| **Survey on choice of intravenous sedative agent at department of dental anesthesiology, Tokyo Dental College Chiba Hospital between 2010 and 2011** | Matsuki, Y; Okamura, T; Shiozaki, K; Matsuura, N; Kasahara, M; Ichinohe, T | 2014 | The Bulletin of Tokyo Dental College | Different outcome |
| **The systemic management of cardiovascular risk patients in dentistry.** | Matsuura, H | 1993 | Anesthesia & pain control in dentistry | Different outcome |
| **Muscle power during intravenous sedation** | Matsuura, N | 2017 | Japanese Dental Science Review | Different outcome |
| **Recognition, assessment and safe management of the medically compromised patient in dentistry** | McCarthy, F M | 1990 | Anesthesia Progress | Different sample |
| **Combination Herbal Therapy (CHT) Versus Placebo in Patients With Irritable Bowel Syndrome (IBS)** | Michael Y Shapira, MD | 2005 | ClinicalTrials.gov | Registration protocol |
| **Dental care for special needs patients: A survey of Texas pediatric dentists** | Milano, M; Seybold, S V | 2002 | Journal of Dentistry for Children | Different outcome |
| **A randomised placebo-controlled trial of the effects of midazolam premedication on children's postoperative cognition** | Millar, K; Asbury, A J; Bowman, A W; Hosey, M T; Martin, K; Musiello, T; Welbury, R R | 2007 | Anaesthesia | Different outcome |
| **Olanzapine Treatment of Adolescent Rats Causes Enduring Specific Memory Impairments and Alters Cortical Development and Function** | Milstein, J A; Elnabawi, A; Vinish, M; Swanson, T; Enos, J K; Bailey, A M; Kolb, B; Frost, D O | 2013 | PLoS ONE | Search noise |
| **Why intravenous moderate sedation should be taught in graduate endodontic programs** | Montagnese, T A | 2012 | Journal of Dental Education | Different type of study |
| **Taurine in management of diffuse cerebral arteriopathy. Clinical and electroencephalographic observations, and mental test results** | Montanini, R; Gasco, P | 1974 | Clinica Terapeutica | Search noise |
| **Assessment of aggressive behaviour in a patient with autism spectrum disorder requiring general anesthesia** | Morrissette, M; Boman, J | 2020 | Journal of the Canadian Academy of Child and Adolescent Psychiatry | Search noise |
| **Bright Hearts: development of a biofeedback controlled interactive artwork for the management of procedural pain and anxiety in children** | Morrow, A; Yogui, M; Poonkhin Khut, G | 2014 | Developmental medicine and child neurology | Different outcome |
| **Medical considerations in dental treatment of children with Williams syndrome** | Moskovitz, M; Brener, D; Faibis, S; Peretz, B | 2005 | Oral Surgery, Oral Medicine, Oral Pathology, Oral Radiology and Endodontology | Different type of study |
| **“I love having benzos after my coke shot”: The use of psychotropic medication among cocaine users in downtown Montreal** | Motta-Ochoa, R; Bertrand, K; Arruda, N; Jutras-Aswad, D; Roy, É | 2017 | International Journal of Drug Policy | Search noise |
| **Provision of psychological support to people in intensive care (v1.0)** | Mouncey, P. | 2015 | ISRCTN Registry | Registration protocol |
| **Marijuana and CF: controversies associated with patient use** | Muirhead, C | 2015 | Pediatric pulmonology | Search noise |
| **Pediatric dentistry and the child with asthma** | Mungo, R P; Kopel, H M; Church, J A | 1986 | Special Care in Dentistry | Search noise |
| **Emergency psychiatry: A review of the literature** | Munizza, C; Furlan, P M; D'Elia, A; D'Onofrio, M R; Leggero, P; Punzo, F; Vidini, N; Villari, V | 1993 | Acta Psychiatrica Scandinavica, Supplement | Different type of study |
| **Treatment of panic disorder** | Muskin, P R; Fyer, A J | 1981 | Journal of Clinical Psychopharmacology | Search noise |
| **Developing a biomarker for anxiety disorders** | N. McNaughton | 2020 | Australian New Zeland Clinical Trials Registry | Registration protocol |
| **A 12 week, multicentre, study investigating the efficacy of ORM-12741 on agitation/aggression symptoms in patients with Alzheimer's Disease** | n.a. | 2015 | EU Clinical Trials Register | Registration protocol |
| **Management of Dental Phobia in Two Outpatients Receiving Fentanyl and Midazolam** | Nakanishi, S; Nagai, N; Shimada, W; Shinohara, A; Fujitaka, W; Kai, Y; Noguchi, D; Goto, T | 2021 | Journal of Japanese Dental Society of Anesthesiology | Different type of study |
| **Sleep-Disordered Breathing and Psychiatric Disorders** | Naqvi, H A; Wang, D; Glozier, N; Grunstein, R R | 2014 | Current Psychiatry Reports | Search noise |
| **Advances in the pharmacotherapy of cognitive deficits and behavioural disorders in dementia** | Naranjo, C A; Best, T S | 1998 | Canadian Journal of Clinical Pharmacology | Search noise |
| **Upregulation of FosB/ΔFosB in limbic circuits after tooth exodontia-induced occlusal instability in an experimental model of unpredictable chronic stress** | Nascimento, G C; de Paula, B B; Ferrari, D P; Iyomasa, D M; Pereira, Y C L; Pedrazzi, J F; Bortolanza, M; Issy, A C; Issa, J P M; Leite-Panissi, C R A; Iyomasa, M M; Del-Bel, E | 2021 | Brain Research Bulletin | Search noise |
| **Behavioral management strategies for young pediatric dental patients with disabilities** | Nathan, J E | 2001 | Journal of Dentistry for Children | Different type of study |
| **Oral Ketamine for the Preinduction of Patients who Aggressively Refuse the Induction of Anaesthesia** | Neckel, W; Jacobs, F E; Tolksdorf, W | 1992 | Anasthesiologie Intensivmedizin Notfallmedizin Schmerztherapie | Abstract not avaible |
| **Opioid and Multi-Drug Pediatric Dental Sedation: A Narrative Review** | Nelson, T; Wilson, S; Tanbonliong, T | 2022 | Journal of Dentistry for Children | Different type of study |
| **Fighting Females: Neural and Behavioral Consequences of Social Defeat Stress in Female Mice** | Newman, E L; Covington III, H E; Suh, J; Bicakci, M B; Ressler, K J; DeBold, J F; Miczek, K A | 2019 | Biological Psychiatry | Search noise |
| **Interest of 50% nitrous oxide and oxygen premix sedation in gerodontology** | Nicolas, E; Lassauzay, C | 2009 | Clinical Interventions in Aging | Different sample |
| **A novel ferritin light chain mutation in neuroferritinopathy with an atypical presentation** | Nishida, K; Garringer, H J; Futamura, N; Funakawa, I; Jinnai, K; Vidal, R; Takao, M | 2014 | Journal of the Neurological Sciences | Search noise |
| **Relationship of insight with medication adherence and the impact on outcomes in patients with schizophrenia and bipolar disorder: Results from a 1-year European outpatient observational study** | Novick, D; Montgomery, W; Treuer, T; Aguado, J; Kraemer, S; Haro, J M | 2015 | BMC Psychiatry | Search noise |
| **Paliperidone for schizophrenia** | Nussbaum, A; Stroup, T S | 2008 | Cochrane Database of Systematic Reviews | Search noise |
| **Anesthetic management during dental treatment in a patient with Aarskog syndrome and a history of technical difficulties during intubation** | Ohiwa, D; Iida, A; Kawahara, K; Honma, S; Kondo, T; Ishida, Y; Ono, S; Fukushima, K | 2017 | Journal of Japanese Dental Society of Anesthesiology | Different type of study |
| **Fluvoxamine versus other anti-depressive agents for depression** | Omori, I M; Watanabe, N; Nakagawa, A; Cipriani, A; Barbui, C; Mcguire, H; Churchill, R; Furukawa, T A | 2010 | Cochrane Database of Systematic Reviews | Search noise |
| **The PACE/PACENET Behavioral Health Laboratory Project** | Oslin, D.W. | 2015 | ClinicalTrials.gov | Registration protocol |
| **Tricyclic antidepressants for attention deficit hyperactivity disorder (ADHD) in children and adolescents** | Otasowie, J; Castells, X; Ehimare, U P; Smith, C H | 2014 | Cochrane Database of Systematic Reviews | Search noise |
| **Addressing health disparities through promoting equity for individuals with intellectual disability** | Ouellette-Kuntz, H; Garcin, N; Lewis, M E S; Minnes, P; Martin, C; Holden, J J A | 2005 | Canadian Journal of Public Health | Search noise |
| **Preferred drug lists: Potential impact on healthcare economics** | Ovsag, K; Hydery, S; Mousa, S A | 2008 | Vascular Health and Risk Management | Search noise |
| **Relation of perceived discomfort to treatment effect in a blinded study of tDCS in older adults with HIV** | Ownby, R L; Rivera, J; Mejia, E; Acevedo, A | 2017 | Brain stimulation | Search noise |
| **International Consensus Statement for the Screening, Diagnosis, and Treatment of Adolescents with Concurrent Attention-Deficit/Hyperactivity Disorder and Substance Use Disorder** | Ozgen, H; Spijkerman, R; Noack, M; Holtmann, M; Schellekens, A S A; Van De Glind, G; Banaschewski, T; Barta, C; Begeman, A; Casas, M; Crunelle, C L; Daigre Blanco, C; Dalsgaard, Sø.; Demetrovics, Z; Den Boer, J; Dom, G; Eapen, V; Faraone, S V; Franck, J; González, R A; Grau-López, L; Groenman, A P; Hemphala, M; Icick, R; Johnson, B; Kaess, M; Kapitány-Fovény, M; Kasinathan, J G; Kaye, S S; Kiefer, F; Konstenius, M; Levin, F R; Luderer, M; Martinotti, G; Matthys, F I A; Meszaros, G; Moggi, F; Munasur-Naidoo, A P; Post, M; Rabinovitz, S; Ramos-Quiroga, J A; Sala, R; Shafi, A; Slobodin, O; Staal, W G; Thomasius, R; Truter, I; Van Kernebeek, M W; Velez-Pastrana, M C; Vollstadt-Klein, S; Vorspan, F; Young, J T; Yule, A; Van Den Brink, W; Hendriks, V | 2022 | Zeitschrift fur Kinder- und Jugendpsychiatrie und Psychotherapie | Search noise |
| **Psychiatric rehabilitation pertaining to health care environments: Facilitating skills and supports of people with mental illness in relation to their mental and physical health care** | Pallaveshi, L; Zisman-Ilani, Y; Roe, D; Rudnick, A | 2013 | Current Psychiatry Reviews | Search noise |
| **Acute severe asthma: New approaches to assessment and treatment** | Papiris, S A; Manali, E D; Kolilekas, L; Triantafillidou, C; Tsangaris, I | 2009 | Drugs | Search noise |
| **Comparison the effect of hypnosis,nitrous oxide and conventional behavior guidance on anxiety level of school-age children during local anesthesia injection and dental extraction** | Parisay, I. | 2019 | Iranian Registry of Clinical Trials | Registration protocol |
| **Pharmacologic agents for smoking cessation: A clinical review** | Patel, D R; Feucht, C; Reid, L; Patel, N D | 2010 | Clinical Pharmacology: Advances and Applications | Search noise |
| **Burning mouth syndrome: A state of the art review for the oral healthcare provider** | Patil, P M; Patil, S P | 2021 | Biomedical and Pharmacology Journal | Search noise |
| **Emotional and affective disorders in epilepsy** | Pauli, E; Stefan, H | 2009 | Nervenarzt | Different type of study |
| **Succinic semialdehyde dehydrogenase deficiency in children and adults** | Pearl, P L; Novomy, E J; Acosta, M T; Jakobs, C; Gibson, K M | 2003 | Annals of Neurology | Search noise |
| **Fetal hazards associated with use of benzodiazepines during the pregnancy: A review** | Peres, R M; Moreira, R K; Moser, C; Berwanger, C G W; Sanseverino, M T V; Schüler-Faccini, L | 2001 | Jornal Brasileiro de Psiquiatria | Different type of study |
| **The use of sedation while treating paediatric dental patients in Israel.** | Peretz, B | 2002 | International journal of paediatric dentistry / the British Paedodontic Society [and] the International Association of Dentistry for Children | Different type of study |
| **Combined cannabinoid therapy via an oromucosal spray** | Pérez, J | 2006 | Drugs of Today | Search noise |
| **PTSD-related behavioral traits in a rat model of blast-induced mtbi are reversed by the MGLUR2/3 receptor antagonist bci-838** | Perez-Garcia, G; de Gasperi, R; Gama Sosa, M A; Perez, G M; Otero-Pagan, A; Tschiffely, A; McCarron, R M; Ahlers, S T; Elder, G A; Gandy, S | 2018 | eNeuro | Search noise |
| **Case series: Acupuncture and pelvic pain** | Pickett, H | 2013 | Medical Acupuncture | Search noise |
| **Development of a screening tool to assess the suitability of people with a disability for oral care under sedation or general anesthesia** | Prabhu, N T; Nunn, J H; Evans, D J; Girdler, N M | 2008 | Special Care in Dentistry | Different outcome |
| **Medical care of adults with mental retardation** | Prater, C D; Zylstra, R G | 2006 | American Family Physician | Different type of study |
| **Transdermal treatment options for neurological disorders: Impact on the elderly** | Priano, L; Gasco, M R; Mauro, A | 2006 | Drugs and Aging | Search noise |
| **Off-label use of risperidone in people with intellectual disability: A discontinuation study** | Ramerman, L. | 2015 | EU Clinical Trials Register | Registration protocol |
| **Orally Disintegrating for the Treatment of Psychotic and Behavioral Disturbances Associated with Dementia** | Reeves, R R; Torres, R A | 2003 | Southern Medical Journal | Search noise |
| **DENTAL CARE FOR HANDICAPPED CHILDREN REEXAMINED: II – DIMENSIONS OF DENTAL PRACTICE** | Roberts, R E; McCrory, O F; Glasser, J H; Askew, C | 1978 | Journal of Public Health Dentistry | Abstract not avaible |
| **Pilot Data on the Feasibility And Clinical Outcomes of a Nomegestrol Acetate Oral Contraceptive Pill in Women With Premenstrual Dysphoric Disorder** | Robertson, E; Thew, C; Thomas, N; Karimi, L; Kulkarni, J | 2021 | Frontiers in Endocrinology | Search noise |
| **Botulinum toxin type A versus anticholinergics for cervical dystonia** | Rodrigues, F B; Duarte, G S; Castelão, M; Marques, R E; Ferreira, J; Sampaio, C; Moore, A P; Costa, J | 2021 | Cochrane Database of Systematic Reviews | Search noise |
| **Drug interactions and adverse reactions** | Roller, L; Gowan, J | 2003 | Australian Journal of Pharmacy | Different outcome |
| **Consent, restraint, and people with special needs: A review** | Romer, M | 2009 | Special Care in Dentistry | Different type of study |
| **Oral Self-Injurious Behaviors in Patients with Developmental Disabilities** | Romer, M; Dougherty, N J | 2009 | Dental Clinics of North America | Search noise |
| **Dentists’ self-perceived stress and difficulties when performing restorative treatment in children** | Rønneberg, A; Strøm, K; Skaare, A B; Willumsen, T; Espelid, I | 2015 | European Archives of Paediatric Dentistry | Search noise |
| **TRIAL OF DRONABINOL ADJUNCTIVE TREATMENT OF AGITATION IN ALZHEIMER'S DISEASE (AD) (THC-AD)** | Rosenberg, P; Forester, B; Agronin, M; Kasckow, J; Amjad, H; Burhanullah, H; Vandrey, R; Outen, J; Skurla, M; May, R; al., et | 2020 | American journal of geriatric psychiatry | Search noise |
| **Distinct cortical thickness correlates of early life trauma exposure and posttraumatic stress disorder are shared among adolescent and adult females with interpersonal violence exposure** | Ross, M C; Sartin-Tarm, A S; Letkiewicz, A M; Crombie, K M; Cisler, J M | 2021 | Neuropsychopharmacology | Search noise |
| **Role of diazepam in dentistry - A short review** | Rubika, J | 2015 | Research Journal of Pharmacy and Technology | Different type of study |
| **Preparedness of Entering Pediatric Dentistry Residents: Advanced Pediatric Program Directors’ and First-Year Residents’ Perspectives** | Rutkauskas, J; Seale, N S; Casamassimo, P; Rutkauskas, J S | 2015 | Journal of Dental Education | Search noise |
| **Maintenance of baroreflex sensitivity during intravenous sedation with a combination of propofol and midazolam** | Sachi, S. | 2010 | UMIN-Clinical Trials Registry | Registration protocol |
| **Anesthetic management of a patient with Costello syndrome** | Sanuki, T; Sugioka, S; Hashimoto, K; Miyano, A; Ishikawa, M; Matsui, H; Ueno, T; Asai, T; Komasa, Y; Kotani, J | 2005 | Journal of Japanese Dental Society of Anesthesiology | Different type of study |
| **Connecting brain proteomics with behavioural neuroscience in translational animal models of neuropsychiatric disorders** | Sarnyai, Z; Guest, P C | 2017 | Advances in Experimental Medicine and Biology | Search noise |
| **Dental care and treatments provided under general anaesthesia in the Helsinki Public Dental Service** | Savanheimo, N; Sundberg, S A; Virtanen, J I; Vehkalahti, M M | 2012 | BMC Oral Health | Search noise |
| **Neurodevelopmental toxicology** | Schmid, C; Rotenberg, J S | 2005 | Neurologic Clinics | Search noise |
| **Drug effects on salivary glands: Dry mouth** | Scully, C | 2003 | Oral Diseases | Search noise |
| **Drug treatment in juvenile depression - Is St. John's wort a safe and effective alternative?** | Seelinger, G; Mannel, M | 2007 | Child and Adolescent Mental Health | Search noise |
| **Paediatric dentistry- novel evolvement** | Shah, S | 2018 | Annals of Medicine and Surgery | Different type of study |
| **Collaborative role of the pediatrician in the diagnosis and management of bipolar disorder in adolescents** | Shain, B N; Braverman, P K; Adelman, W P; Breuner, C C; Levine, D A; Marcell, A V; Murray, P J; O'Brien, R F | 2012 | Pediatrics | Search noise |
| **Neuroprotective effect of 25-Methoxyhispidol A against CCl4-induced behavioral alterations by targeting VEGF/BDNF and caspase-3 in mice** | Shal, B; Khan, A; Naveed, M; Ali, H; Seo, E K; Choi, H; Khan, S | 2020 | Life Sciences | Search noise |
| **The clinical activity of a new benzodiazepin: cloxazolam** | Singer, L; Krieger-Finance, F; Danion, J M | 1980 | Annales Medico-Psychologiques | Different type of study |
| **Comparison of Remifentanil as a Sole Agent or in Combination With Midazolam Versus Fentanyl/Midazolam During Sedation for Colonoscopy** | Singh, R. | 2015 | ClinicalTrials.gov | Registration protocol |
| **Treatment of Mentally Disabled Patients with Intravenous Sedation in a Dental Clinic Outpatient Setting** | Solomowitz, B H | 2009 | Dental Clinics of North America | Different type of study |
| **Depression in children and adolescents** | Son, S E; Kirchner, J T | 2000 | American Family Physician | Search noise |
| **Brazil before and during covid-19 pandemic: Impact on the practice and habits of physical exercise** | Sonza, A; de Sá-Caputo, D D C; Bachur, J A; de Araújo, M.D.G.R.; Trippo, K V; da Gama, D R N; Borges, D L; Mendonça, V A; Bernardo-Filho, M | 2021 | Acta Biomedica | Search noise |
| **Maxillary Sinus Floor Augmentation in Diabetic Patients** | Starch-Jensen, S. | 2020 | ClinicalTrials.gov | Registration protocol |
| **Sinus Floor Augmentation and Graft Compared Sinus Membrane Elevation With Blood Coagulum. A Randomized Controlled Trial** | Starch-Jensen, T. | 2020 | ClinicalTrials.gov | Registration protocol |
| **Adult minimal oral sedation in the general practice setting** | Stillwell, K D; Anderson, B J | 2012 | General Dentistry | Search noise |
| **The atease study: a multicenter randomized clinical trial of the safety and efficacy of TNX-102 Sl in the treatment of military-related PTSD** | Sullivan, G; Gendreau, J; Michael Gendreau, R; Schaberg, A; Daugherty, B; Jividen, H; Peters, A; Peters, P; Weathers, F; Lederman, S | 2016 | Neuropsychopharmacology | Search noise |
| **Study of adverse drug effects of antiepileptic drugs used in pediatric patients in a tertiary care rural hospital-a pharmacovigilance study** | Suman, A; Gosavi, D D | 2017 | Journal of Young Pharmacists | Search noise |
| **Alzheimer's disease and oral health** | Sumer, A P; Sumer, M | 2005 | Neurosciences | Abstract not avaible |
| **Glycine transporter 1 inhibitors and modulation of NMDA receptor-mediated excitatory neurotransmission** | Sur, C; Kinney, G G | 2007 | Current Drug Targets | Search noise |
| **The pediatric role in the care of children in foster and kinship care** | Szilagyi, M | 2012 | Pediatrics in Review | Search noise |
| **Reply to Snow et al. regarding their comment ‘A call to solve the puzzle together by building an evidence base for perioperative management of children with Autism Spectrum Disorder (ASD)** | Taghizadeh, N; Davidson, A; Williams, K; Story, D | 2016 | Paediatric Anaesthesia | Abstract not avaible |
| **Systemic management during dental treatment of a patient with cerebral infarction due to antiphospholipid syndrome (APS)** | Takagi, J; Rakugi, M; Nishida, M; Shibutani, T; Niwa, H; Kim, Y; Matsuura, H | 1997 | Journal of Japanese Dental Society of Anesthesiology | Different type of study |
| **Anesthetic management of a patient with mental disabilities as well as the past history of heat stroke and neuroleptic malignant syndrome** | Takaishi, K; Nakajo, N | 2007 | Japanese Journal of Anesthesiology | Different outcome |
| **St8sia2 deficiency plus juvenile cannabis exposure in mice synergistically affect higher cognition in adulthood** | Tantra, M; Kröcher, T; Papiol, S; Winkler, D; Röckle, I; Jatho, J; Burkhardt, H; Ronnenberg, A; Gerardy-Schahn, R; Ehrenreich, H; Hildebrandt, H | 2014 | Behavioural Brain Research | Search noise |
| **General anaesthesia of out-patients in pedodontics.** | Tarján, I; Mikecz, G; Dénes, J | 1990 | Journal of the International Association of Dentistry for Children | Different outcome |
| **Comparison of oral and buccal midazolam for pediatric dental sedation** | Tavassoli, S. | 2013 | Iranian Registry of Clinical Trials | Registration protocol |
| **Early treatment of acute stress disorder in children with major burn injury** | Tcheung, W J; Robert, R; Rosenberg, L; Rosenberg, M; Villarreal, C; Thomas, C; Holzer III, C E; Meyer III, W J | 2005 | Pediatric Critical Care Medicine | Search noise |
| **Intravenous Sedation with Midazolam, Dexmedetomidine, and Fentanyl during the Extraction of Impacted Wisdom Teeth in a Patient with Dental Phobia** | Teshirogi, T; Takuma, S; Hashimoto, K; Hojo, T; Hase, Y; Fujisawa, T | 2021 | Journal of Japanese Dental Society of Anesthesiology | Different type of study |
| **Neuropsychopharmacology of a therapeutically used Andrographis paniculata extract: A preclinical study** | Thakur, A K; Chatterjee, S S; Kumar, V | 2014 | Oriental Pharmacy and Experimental Medicine | Search noise |
| **The MAGIC trial (Melatonin for Anxiety prior to General anaesthesia In Children)** | Thomason, A. L. | 2019 | EU Clinical Trials Register | Registration protocol |
| **GABAAReceptors and Maternally Derived Taurine Regulate the Temporal Specification of Progenitors of Excitatory Glutamatergic Neurons in the Mouse Developing Cortex** | Tochitani, S; Furukawa, T; Bando, R; Kondo, S; Ito, T; Matsushima, Y; Kojima, T; Matsuzaki, H; Fukuda, A | 2021 | Cerebral Cortex | Search noise |
| **Physical health, behavioral and emotional functioning in children of gulf war veterans** | Toomey, R; Alpern, R E; White, A J; Li, X; Reda, D J; Blanchard, M S | 2021 | Life Sciences | Search noise |
| **Efficacy and Safety Study of a IMSS Developed Phytopharmaceutical for the Treatment of Anxiety** | Tortoriello, J. | 2018 | ClinicalTrials.gov | Registration protocol |
| **Medication compliance, adherence, and persistence: Current status of behavioral and educational interventions to improve outcomes** | Touchette, D R; Shapiro, N L | 2008 | Journal of Managed Care Pharmacy | Search noise |
| **Generalised anxiety disorder** | Tyrer, P; Baldwin, D | 2006 | Lancet | Search noise |
| **Anxiety, depression, and diseases of the lower extremities** | Udell, E T; Weiss, K J | 1998 | Clinics in Podiatric Medicine and Surgery | Search noise |
| **Neuroprotection after traumatic brain injury in heat-acclimated mice involves induced neurogenesis and activation of angiotensin receptor type 2 signaling** | Umschweif, G; Shabashov, D; Alexandrovich, A G; Trembovler, V; Horowitz, M; Shohami, E | 2014 | Journal of Cerebral Blood Flow and Metabolism | Search noise |
| **Bariatric Endoscopy for Treatment of I and II Grade Obesity** | Vadalà di Prampero, S.F. | 2021 | ClinicalTrials.gov | Registration protocol |
| **Conscious sedation with midazolam in a dental patient with a spastic nerve/muscle disorder--a case report.** | van der Bijl, P; Roelofse, J A | 1994 | Annals of dentistry | Different type of study |
| **Potentiation of responses to AMPA on central neurones by LY392098 and LY404187 in vivo** | Vandergriff, J; Huff, K; Bond, A; Lodge, D | 2001 | Neuropharmacology | Search noise |
| **Development of a new tool for predicting the behavior of individuals with intellectual disability in the dental office: A pilot study** | Varela, I; Fernández-Feijoo, J; García, E; Diniz-Freitas, M; Martínez, I; Roca, J; Diz, P; Limeres, J | 2022 | Disability and Health Journal | Different type of study |
| **Strategies for addressing adherence problems in patients with serious and persistent mental illness: Recommendations from the expert consensus guidelines** | Velligan, D I; Weiden, P J; Sajatovic, M; Scott, J; Carpenter, D; Ross, R; Docherty, J P | 2010 | Journal of Psychiatric Practice | Search noise |
| **Oral risperidone, olanzapine and quetiapine versus haloperidol in psychotic agitation** | Villari, V; Rocca, P; Fonzo, V; Montemagni, C; Pandullo, P; Bogetto, F | 2008 | Progress in neuro-psychopharmacology & biological psychiatry | Search noise |
| **Release phenomena and iterative activities in psychiatric geriatric patients** | Villeneuve, A; Turcotte, J; Bouchard, M; Côté, J M; Jus, A | 1974 | Canadian Medical Association Journal | Search noise |
| **Homer1/mGluR5 Activity Moderates Vulnerability to Chronic Social Stress** | Wagner, K V; Hartmann, J; Labermaier, C; Häusl, A S; Zhao, G; Harbich, D; Schmid, B; Wang, X.-D.; Santarelli, S; Kohl, C; Gassen, N C; Matosin, N; Schieven, M; Webhofer, C; Turck, C W; Lindemann, L; Jaschke, G; Wettstein, J G; Rein, T; Müller, M B; Schmidt, M V | 2015 | Neuropsychopharmacology | Search noise |
| **Testosterone has antidepressant-like efficacy and facilitates imipramine-induced neuroplasticity in male rats exposed to chronic unpredictable stress** | Wainwright, S R; Workman, J L; Tehrani, A; Hamson, D K; Chow, C; Lieblich, S E; Galea, L A M | 2016 | Hormones and Behavior | Search noise |
| **Non-vision adverse events with vigabatrin therapy** | Walker, S D; Kälviäinen, R | 2011 | Acta Neurologica Scandinavica | Search noise |
| **Sexual dysfunction in the older woman: An overview of the current understanding and management** | Walsh, K E; Berman, J R | 2004 | Drugs and Aging | Search noise |
| **The proceedings of the 15th professional conference on Williams Syndrome** | Walton, J R; Martens, M A; Pober, B R | 2017 | American Journal of Medical Genetics, Part A | Search noise |
| **Should Canadian patients look forward to aducanumab for Alzheimer disease?** | Watt, J A; Marple, R; Hemmelgarn, B; Straus, S E | 2021 | CMAJ | Different sample |
| **Efficacy and Safety of Lanabecestat for Treatment of Early and Mild Alzheimer Disease: the AMARANTH and DAYBREAK-ALZ Randomized Clinical Trials** | Wessels, A M; Tariot, P N; Zimmer, J A; Selzler, K J; Bragg, S M; Andersen, S W; Landry, J; Krull, J H; Downing, A M; Willis, B A; al., et | 2020 | JAMA neurology | Search noise |
| **Clinical effectiveness, tolerability and cost-effectiveness of newer drugs for epilepsy in adults: A systematic review and economic evaluation** | Wilby, J; Kainth, A; Hawkins, N; Epstein, D; McIntosh, H; McDaid, C; Mason, A; Golder, S; O'Meara, S; Sculpher, M; Drummond, M; Forbes, C | 2005 | Health Technology Assessment | Search noise |
| **Efficacy of Hypnosis on Dental Anxiety and Phobia: A Systematic Review and Meta-Analysis** | Wolf, T G; Schläppi, S; Benz, C I; Campus, G | 2022 | Brain Sciences | Search noise |
| **Anesthesia for special needs - Small infants** | Wong, T.K.-M. | 2012 | Acta Anaesthesiologica Taiwanica | Search noise |
| **The interpersonal work of dental conscious sedation: A qualitative analysis** | Woolley, S M; Chadwick, B; Pugsley, L | 2017 | Community Dentistry and Oral Epidemiology | Different outcome |
| **SAAD annual conference. 31st October 1998. Royal Society of Medicine, London.** | Wraith, A | 1999 | SAAD digest | Different type of study |
| **Association of Dental Anaesthetists. Summer Scientific Meeting Stirling, Scotland 8-9 June, 2001. ADA meeting report.** | Wraith, A | 2001 | SAAD digest | Different type of study |
| **Making patients safe and comfortable for a lifetime of dentistry: frontiers in office-based sedation.** | Yagiela, J A | 2001 | Journal of dental education | Different type of study |
| **Changes in heart rate during administration of local anesthetics in neuropsychiatric patients** | Yakata, H; Yokobayashi, T; Nakajima, T | 1979 | Journal of Oral Surgery | Different outcome |
| **Mask Induction for an Intellectually Disabled Patient With Congenital Infiltrating Lipomatosis of the Face** | Yamanaka, H; Tsukamoto, M; Hitosugi, T; Yokoyama, T | 2020 | Anesthesia progress | Different type of study |
| **Whole exome sequencing identifies a novel predisposing gene, MAPKAP1, for familiar mixed mood disorder** | Yang, C; Li, S; Ma, J X; Li, Y; Zhang, A; Sun, N; Wang, Y; Xu, Y; Zhang, K | 2019 | Frontiers in Genetics | Search noise |
| **Mechanistic Target of Rapamycin–Independent Antidepressant Effects of (R)-Ketamine in a Social Defeat Stress Model** | Yang, C; Ren, Q; Qu, Y; Zhang, J.-C.; Ma, M; Dong, C; Hashimoto, K | 2018 | Biological Psychiatry | Search noise |
| **Root canal treatment and special needs patients** | Yap, E; Parashos, P; Borromeo, G L | 2015 | International Endodontic Journal | Different outcome |
| **A retrospective analysis of dental procedures performed under general anesthesia** | Yilmaz, Z; Isik, B | 2016 | Anestezi Dergisi | Search noise |
| **Behavioral response to fluoxetine in both female and male mice is modulated by dentate gyrus granule cell activity** | Yohn, C N; Dieterich, A; Maita, I; Bazer, A S; Diethorn, E; Ma, D; Gergues, M M; Hu, P; Samuels, B A | 2020 | Neurobiology of Stress | Search noise |
| **Adherence to therapy in psychiatric patients: an empirical investigation** | Youssef, F A | 1984 | International Journal of Nursing Studies | Search noise |
| **Effect of propranolol on facial scanning in autism spectrum disorder: a preliminary investigation** | Zamzow, R M; Christ, S E; Saklayen, S S; Moffitt, A J; Bodner, K E; Higgins, K F; Beversdorf, D Q | 2014 | Journal of clinical and experimental neuropsychology | Search noise |
| **Hormonal contraception increases the risk of psychotropic drug use in adolescent girls but not in adults: A pharmacoepidemiological study on 800 000 Swedish women** | Zettermark, S; Vicente, R P; Merlo, J | 2018 | PLoS ONE | Search noise |
| **The clinical and experimental research on the treatment of anxiety with An Shenfang** | Zhang, X | 2021 | Dissertation for doctoral degree of southern medical university [nanfang yike daxue boshi xuewei lunwen] | Search noise |
| **Ketamine administered to pregnant rats in the second trimester causes long-lasting behavioral disorders in offspring** | Zhao, T; Li, Y; Wei, W; Savage, S; Zhou, L; Ma, D | 2014 | Neurobiology of Disease | Search noise |
| **True Functional Restoration and Analgesia in Non-Radicular Low Back Pain** | Zimmerman, A.A. | 2022 | ClinicalTrials.gov | Registration protocol |
| **Assessment of Family Based Intervention Needs by a Direct Survey of Patients with a Psychiatric Disorder** | Zogas, K; Juckel, G; Mavrogiorgou, P | 2020 | Psychiatrische Praxis | Search noise |

Table S4. List of articles added after consulting reference lists.

| **Title** | **Authors** | **Published Year** | **Journal** |
| --- | --- | --- | --- |
| **Nitrous oxide dosage in relative analgesia.** | Allen WA. | 1984 | British Dental Journal |
| **Conscious sedation procedures using intravenous midazolam for dental care in patients with different cognitive profiles: a prospective study of effectiveness and safety.** | Collado V, Faulks D, Nicolas E, Hennequin M. | 2013 | PLoS One |
| **Autistic patients: a retrospective study on their dental needs and the behavioural approach.** | Mangione F, Bdeoui F, Monnier-Da Costa A, Dursun E. | 2020 | Clinical Oral Investigation |
| **Risk factors with intravenous sedation for patients with disabilities.** | Yoshikawa F, Tamaki Y, Okumura H, Miwa Z, Ishikawa M, Shimoyama K, Nakamura Z, Kunimori H, Jinno S, Kohase H, Fukayama H. | 2013 | Anesthesia Progress |

Table S5. Excluded articles and reason of exclusion after full text analysis.

| **Title** | **Authors** | **Published Year** | **Journal** | **Reason of exclusion** |
| --- | --- | --- | --- | --- |
| **Administration of intravenous sedation with midazolam by dentists is unsafe** | Broers, D L; Plat, J; de Jongh, A; Zuidgeest, T G; Blom, H C; Kraaijenhagen, A E; Pieterse, C M; Bildt, M M | 2015 | Nederlands tijdschrift voor tandheelkunde | Different outcomes |
| **Orthognathodontic therapy in handicapped patients** | Celli, D; Polisini, J C; D'Addona, A | 1990 | Minerva ortognatodontica | Different study design |
| **Intravenous sedation: An adjunct to enable orthodontic treatment for children with disabilities** | Chaushu, S; Gozal, D; Backer, A | 2002 | European Journal of Orthodontics | Different outcomes |
| **Neurosedation in dentistry of the disabled patient: the use of midazolam, propofol, and remifentanil.** | Collini, S; Pinto, G; Lejeune, L; Di Carlo, S; Meloncelli, S; Barraco, G; Gatto, R | 2006 | Minerva stomatologica | Different outcomes |
| **Comparison of the sedative effectiveness of two pharmacological regimens.** | Davila, J M; Herman, A E; Proskin, H M; Vitale, D | 1994 | ASDC journal of dentistry for children | Different study design |
| **Behavior guidance techniques used in dental care for patients with special needs: Acceptance of parents** | de Castro, A M; Espinosa, R C G; Pereira, C A M; Castro, T C; Santos, M.A.S.B.; Santos, D R; Oliveira, F S | 2016 | Pesquisa Brasileira em Odontopediatria e Clinica Integrada | Different outcomes |
| **Clinical effectiveness of inhalation conscious sedation with nitrous oxide and oxygen for dental treatment in uncooperative paediatric patients during COVID-19 outbreak** | Ferrazzano, G F; Quaraniello, M; Sangianantoni, G; Ingenito, A; Cantile, T | 2020 | European Journal of Paediatric Dentistry | Different sample |
| **The sedative effect of intranasal midazolam administration in the dental treatment of patients with mental disabilities. Part 1. The effect of a 0.2 mg/kg dose.** | Fukuta, O; Braham, R L; Yanase, H; Atsumi, N; Kurosu, K | 1993 | The Journal of clinical pediatric dentistry | Different study design |
| **The sedative effects of intranasal midazolam administration in the dental treatment of patients with mental disabilities. Part 2: optimal concentration of intranasal midazolam.** | Fukuta, O; Braham, R L; Yanase, H; Kurosu, K | 1994 | The Journal of clinical pediatric dentistry | Different study design |
| **Clinical effects of training in behavior management on handicapped patients in dentistry** | Fukuta, O; Ohishi, N; Suzuki, Y; Maruyama, H; Yanase, H; Kurosu, K | 1989 | Shoni shikagaku zasshi. The Japanese journal of pedodontics | Not in english |
| **Premedication with midazolam in intellectually disabled dental patients: Intramuscular or oral administration? A retrospective study** | Hanamoto, H; Boku, A; Sugimura, M; Oyamaguchi, A; Inoue, M; Niwa, H | 2016 | Medicina Oral Patologia Oral y Cirugia Bucal | Different protocol of administration |
| **The influence of clonidine on oral ketamine-midazolam premedication in intellectually disabled patients indicated for dental procedures: Double-blind comparison of two sedation regimes** | Horacek, J; Palenicek, T; Malek, J; Scigel, V; Kurzova, A; Hess, L | 2012 | Neuroendocrinology Letters | Different outcomes |
| **Dental treatment of handicapped patients with conscious sedation** | Jakobs, W; Lipp, M; Daubländer, M; Jakobs-Hannegrefs, E | 1989 | Anesthesia Progress | Different study design |
| **Comparison of sevoflurane and propofol's effects on hemodynamics and recovery in mentally retarded pediatric patients undergoing outpatient dental surgery** | Kocabaş, S; Yüksel, E; Yediçocuklu, D; Aşkar, F Z; Balcioǧlu, T; Koca, H | 2014 | Turkiye Klinikleri Journal of Medical Sciences | Not in english |
| **Accumulated oropharyngeal water increases coughing during dental treatment with intravenous sedation** | Kohjitani, A; Egusa, M; Shimada, M; Miyawaki, T | 2008 | Journal of Oral Rehabilitation | Different sudy design |
| **A study of painless local anesthesia: effects of topical anesthetics and nitrous oxide** | Koshiba, K; Kasahara, H; Hosaka, K; Ogasawara, T; Watanabe, T | 1995 | Journal of japanese dental society of anesthesiology | Different outcomes |
| **Pharmacological behaviour support for adults with intellectual disabilities: Frequency and predictors in a national cross-sectional survey** | Mac Giolla Phadraig, C; Griffiths, C; McCallion, P; McCarron, M; Donnelly-Swift, E; Nunn, J | 2018 | Community Dentistry and Oral Epidemiology | Different outcomes |
| **Nitrous-oxide sedation indispensable in the dental care of anxious people and the mentally impaired** | Makkes, P C; Jonker, M J; Turk, T | 2006 | Nederlands Tijdschrift voor Geneeskunde | Not in english |
| **Inhalation sedation in dentistry** | Makkes, P C; Vermeulen-Cranch, D M E; De Zeeuw-Van Gerven, M; Thoden Van Velzen, S K | 1981 | Nederlands Tijdschrift voor Geneeskunde | Not in english |
| **Autistic patients: a retrospective study on their dental needs and the behavioural approach.** | Mangione F, Bdeoui F, Monnier-Da Costa A, Dursun E. | 2020 | Clinical Oral Investigations | Different outcomes |
| **Intravenous sedation for dental patients with intellectual disability** | Miyawaki, T; Kohjitani, A; Maeda, S; Egusa, M; Mori, T; Higuchi, H; Kita, F; Shimada, M | 2004 | Journal of Intellectual Disability Research | Different study design |
| **Comparison of nitrous-oxide oxygen as inhalation agent, Midazolam, Ketamine alone and in combination as oral sedative agents for in-office paediatric patients- a randomised control trial** | Nagpal, M; Khatri, M L; Gupta, A; Srivastava, A; Garg, S | 2021 | Journal of clinical and diagnostic research | Different sample |
| **Conscious sedation with propofol in dentistry** | Oei-Lim, L B; Vermeulen-Cranch, D M; Bouvy-Berends, E C | 1991 | British Dental Journal | Different sample |
| **Conscious sedation in dentistry using Propofol** | Oei-Lim, L B; Vermeulen-Cranch, D M; Bouvy-Berends, E C | 1990 | Nederlands Tijdschrift voor Tandheelkunde | Not in english |
| **Computer controlled infusion of propofol for conscious sedation in dental treatment** | Oei-Lim, V L B; Kalkman, C J; Makkes, P C; Ooms, W G; Hoogstraten, J | 1997 | British Dental Journal | Different sample |
| **Availability of hospital dental care services under sedation or general anesthesia for individuals with special needs in the unified health system for the state of minas Gerais (SUS-MG), Brazil** | Santos, J S; Valle, D A; Palmier, A C; do Amaral, J H L; de Abreu, M.H.N.G. | 2015 | Ciencia e Saude Coletiva | Different study design |
| **Combined use of low-dose propofol and sevoflurane anesthesia for dental treatment in severely disabled patients** | Shiki, A; Ishikura, Y; Ogata, K | 2009 | Journal of Japanese Dental Society of Anesthesiology | Not in english |
| **Factors affecting rectal temperature during anesthesia for dental treatment in mentally disabled patients** | Shiki, A; Ishikura, Y; Ogata, K | 2010 | Journal of Japanese Dental Society of Anesthesiology | Not in english |
| **Oral rehabilitation and management of mentally retarded** | Solanki, J; Khetan, J; Gupta, S; Tomar, D; Singh, M | 2015 | Journal of Clinical and Diagnostic Research | Different outcomes |
| **Anesthetic management of dental procedures in patients with special needs: A retrospective analysis of 519 patients in North of Turkey** | Yilmaz, M Z; Torun, A C; Baş, B; Duran, H; Köse, H I; Furuncuoʇlu, H | 2014 | Journal of Experimental and Clinical Medicine (Turkey) | Different study design |
| **Risk factors with intravenous sedation for patients with disabilities.** | Yoshikawa F, Tamaki Y, Okumura H, Miwa Z, Ishikawa M, Shimoyama K, Nakamura Z, Kunimori H, Jinno S, Kohase H, Fukayama H. | 2013 | Anesthesia Progress | Different protocol of administration |
| **Nitrous oxide dosage in relative analgesia.** | Allen WA. | 1984 | British Dental Journal | Different study design |
